# An embedded interfacial network stabilizes inorganic CsPbI_3_ perovskite thin films

**DOI:** 10.1038/s41467-022-35255-9

**Published:** 2022-12-06

**Authors:** Julian A. Steele, Tom Braeckevelt, Vittal Prakasam, Giedrius Degutis, Haifeng Yuan, Handong Jin, Eduardo Solano, Pascal Puech, Shreya Basak, Maria Isabel Pintor-Monroy, Hans Van Gorp, Guillaume Fleury, Ruo Xi Yang, Zhenni Lin, Haowei Huang, Elke Debroye, Dmitry Chernyshov, Bin Chen, Mingyang Wei, Yi Hou, Robert Gehlhaar, Jan Genoe, Steven De Feyter, Sven M. J. Rogge, Aron Walsh, Edward H. Sargent, Peidong Yang, Johan Hofkens, Veronique Van Speybroeck, Maarten B. J. Roeffaers

**Affiliations:** 1 cMACS, Department of Microbial and Molecular Systems, KU Leuven, 3001 Leuven, Belgium; 2 Department of Chemistry, University of California, Berkeley, CA 94720 USA; 3 School of Mathematics and Physics, The University of Queensland, Brisbane, QLD 4072 Australia; 4 Center for Molecular Modeling (CMM), Ghent University, Technologiepark 46, 9052 Zwijnaarde, Belgium; 5 Department of Chemistry, KU Leuven, Celestijnenlaan 200F, Leuven, 3001 Belgium; 6 Department of Electrical and Computer Engineering, University of Toronto, 35 St George Street, Toronto, Ontario M5S 1A4 Canada; 7 NCD-SWEET beamline, ALBA synchrotron light source, 08290 Cerdanyola del Vallès, Barcelona Spain; 8 CEMES/CNRS, Université de Toulouse, 29, rue Jeanne Marvig, 31055 Toulouse, France; 9 IMEC, Kapeldreef 75, 3001 Leuven, Belgium; 10 Department of Electrical Engineering (ESAT), KU Leuven, Kasteelpark Arenberg 10, 3001 Leuven, Belgium; 11 The Molecular Foundry, Lawrence Berkeley National Laboratory, Berkeley, California 94720 USA; 12 Materials Sciences Division, Lawrence Berkeley National Laboratory, Berkeley, CA 94720 USA; 13 Department of Materials Science and Engineering, University of California, Berkeley, CA 94720 USA; 14 Swiss-Norwegian Beamlines at the European Synchrotron Radiation Facility, 71 Avenue des Martyrs, F-38000 Grenoble, France; 15 Department of Materials, Imperial College London, Exhibition Road, London, SW7 2AZ United Kingdom; 16 Department of Materials Science and Engineering, Yonsei University, Seoul, 120-749 Korea; 17 Kavli Energy Nano Science Institute, Berkeley, CA 94720 USA; 18 Max Plank Institute for Polymer Research, Mainz, D−55128 Germany

**Keywords:** Solar cells, Phase transitions and critical phenomena, Chemical engineering

## Abstract

The black perovskite phase of CsPbI_3_ is promising for optoelectronic applications; however, it is unstable under ambient conditions, transforming within minutes into an optically inactive yellow phase, a fact that has so far prevented its widespread adoption. Here we use coarse photolithography to embed a PbI_2_-based interfacial microstructure into otherwise-unstable CsPbI_3_ perovskite thin films and devices. Films fitted with a tessellating microgrid are rendered resistant to moisture-triggered decay and exhibit enhanced long-term stability of the black phase (beyond 2.5 years in a dry environment), due to increasing the phase transition energy barrier and limiting the spread of potential yellow phase formation to structurally isolated domains of the grid. This stabilizing effect is readily achieved at the device level, where unencapsulated CsPbI_3_ perovskite photodetectors display ambient-stable operation. These findings provide insights into the nature of phase destabilization in emerging CsPbI_3_ perovskite devices and demonstrate an effective stabilization procedure which is entirely orthogonal to existing approaches.

## Abstract (web summary)

Lattice anchoring, in its varied forms, has proven effective at regulating the energetics of metastable phases of polymorphic crystals. Here, the authors utilize top-down photolithography to embed a tessellating 3D interfacial network into otherwise-unstable CsPbI_3_ perovskite thin films and devices, stabilizing the perovskite phase.

## Introduction

**Fig. 1:**
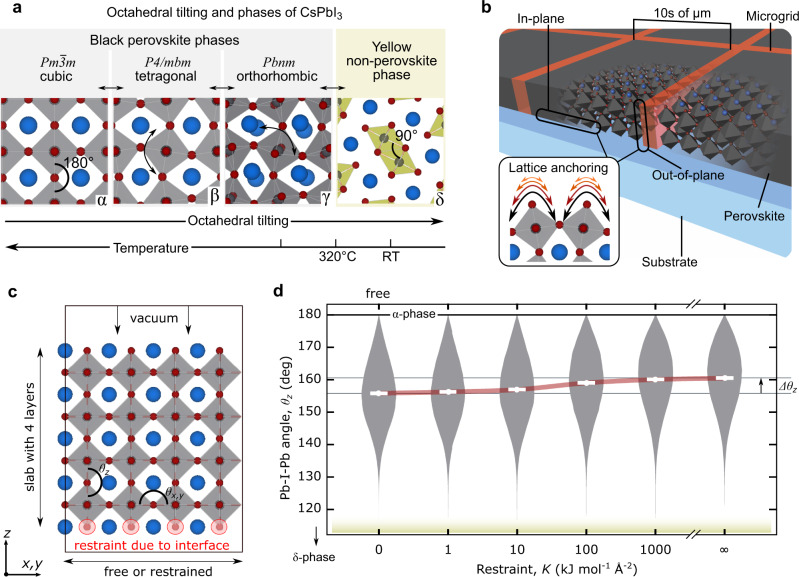
Phase transitions and lattice anchoring in CsPbI_3_. **a** CsPbI_3_ crystal phases represented as a general function of temperature and octahedral tilting (indicated using curved arrows). Pb atoms are in black, I in red, and Cs in blue. **b** Illustration of anchoring imposed by a microgrid introduced into a thin film. **c** Initial position of a molecular dynamics (MD) simulation of a 4 x 4 x 4 supercell with I^−^ anions at the bottom (circled in red) restrained by a harmonic bias potential, *V*(*d*) *=* *Kd²*, with *d* being the distance from their initial position and the *K* the strength of the restraint. **d** Probability distributions (grey) and averages (white) of all *θ*_*z*_ bond angles during the MD simulations as a function of *K*. The red line connecting the averages values is a guide for the eye. The effect of increasing *K* converges near 1000 kJ mol^−1^ Å^−2^, which is the typical order of magnitude of covalently bonded atoms.

The technological progress and widespread adoption of high-efficiency organic-inorganic lead halide perovskite solar cells^1^ are limited by their instability under ambient conditions^2,3^. The chemical volatility associated with the use of organic cations in methylammonium and formamidinium lead iodide perovskites is mitigated using Cs^4^, resulting in a chemically more stable all-inorganic CsPbI_3_ perovskite, which is capable of making efficient solar cell devices^5,6^. At room temperature (RT), bulk CsPbI_3_ forms a stable yellow non-perovskite crystal structure (δ) and requires temperatures above 320 °C to form a perovskite phase^7^ (α, β, and γ: Fig. 1a). When a bulk CsPbI_3_ material is cooled from the high-temperature α-phase, Pb-I-Pb bond angles distort to preserve a corner-sharing structure (~180°) before bond breaking occurs, restructuring into the δ-phase (~90°).

Under ambient conditions, exposure to atmospheric moisture is a central issue leading to phase destabilization of CsPbI_3_ perovskite at room temperature. Unlike the chemical decomposition of organic-inorganic hybrid perovskites, H_2_O molecules adsorb onto the CsPbI_3_ surface and partially dissolve the halide anions^8^, creating vacancy defect sites that lower the phase transition energy barrier between the black γ-phase and the yellow δ-phase. Subsequently, changes in the relative humidity lead directly to an exponential increase in the vacancy concentration – rapidly increasing the yellow phase nucleation rate^9^. There are several ways to shift the phase energetics of CsPbI_3_^10^ in favor of the perovskite phase at ambient conditions – without adjusting its composition – with lattice anchoring proving successful in several implementations: surface functionalization^11^ and (nano)confinement^12,13^, as well as planar interface engineering^14^. Compared to the facile solution-processing methods which have made halide perovskites so appealing^15^, these approaches can complicate device designs, e.g., by either interrupting the bulk optoelectronic properties of the perovskite or by imposing limits on the high-temperature processing. After annealing a typical solution-processed CsPbI_3_ thin film, strain is introduced into the perovskite crystal at the substrate interface due to a thermal expansion mismatch^16^, which improves the stability of the perovskite^17^ (Supplementary Note 1). The 2D interface itself will provide lattice anchoring sites which helps stabilize the perovskite phase, however, this effect remains limited to the planar region where the materials join. Unlike crystal strain which primarily shifts the relative formation energies of the competing phases^16^, lattice anchoring is expected to specifically raise the phase transition energy barrier^10^ and the two can be combined to secure a more ambient-stable perovskite structure.

Here we embed a 3D network of anchoring sites into CsPbI_3_ thin films to bolster the interfacial effect throughout the film and reinforce the desired, functional, perovskite phase. We construct a tessellating microgrid (Fig. 1b) to form isolated micrometer domains of perovskite and we form films made up of these regions. We employ photolithography^18^ to the pattern, using top-down (photo)thermal conversion of CsPbI_3_ into local endogenous deposits of PbI_2_. Beyond merely introducing a periodic lattice anchoring structure, the black-to-yellow phase transformation is shown to be a nucleation limited reaction and the overall stability of processed films is greatly enhanced by preventing the nucleation events from spreading beyond a single region of the grid. Compared to as-grown solution processed CsPbI_3_ perovskite thin films (Supplementary Fig. 1), which complete their black-to-yellow phase transitions in under 10 hours within a dry environment (i.e. a strained black thin film acting as a control:^16^ see Supplementary Figs. 2 and 3), perovskite thin films embedded with a microgrid persist beyond 2.5 years when shielded from moisture. Using perovskite photodetectors as a conceptual demonstrator, we show how this approach offers a simple and effective strategy toward ambient processed and stable CsPbI_3_ optical devices.

## Results

### Simulating the crystal structure under induced lattice anchoring

We begin with an examination of how lattice anchoring stabilizes the CsPbI_3_ black phase by performing molecular dynamics (MD) simulations (expanded discussion on the computational and analytical approaches used can be found in the Supplementary Note 2). Considering a 4 × 4 × 4 supercell of a slab with 4 layers of CsPbI_3_ (Supplementary Fig. 4), the Pb-I-Pb bond angles are separated in 3 groups; *θ*_*x*_, *θ*_*y*_, and *θ*_*z*_. These are defined relative to the in-plane (*x* and *y*) restrictions applied to the anions during the MD simulation, and out-of-plane relaxation direction (*z*). Applying different degrees of planar restraint (Fig. 1c), the running average of the Pb-I-Pb bond angles (Supplementary Fig. 5) quickly decreases from 180° (an initial α-phase) to approximately 160° (γ-phase), forming varied distributions depending on the restraint. The simulated interface is found to predominantly impact out-of-plane bond angles (*θ*_z_; Supplementary Fig. 6) and skew their distributions upward (Supplementary Fig. 7) with increasing restraint strength, *K* (Fig. 1d). To mimic lattice anchoring while strained due to a thermal expansion mismatch with the substrate, lattice vectors were further constrained to their value at annealing temperatures (600 K), relative to a simulated return to RT (300 K), showing that the angles increase for both the strained in-plane directions (*θ*_*x*_ and *θ*_*y*_) and for the perpendicular, out-of-plane direction (*θ*_z_; Supplementary Fig. 8). These findings indicate that, while anchored, additional bending energy is required to transform from the perovskite phase to the yellow δ-phase (Supplementary Fig. 9). For large *K* values, the effect resembles the epitaxial growth of metastable materials using substrates with different crystal symmetry and lattice constants, i.e., even though the restricted geometry of the first unit cell relaxes away from interface (Supplementary Fig. 10), the restraint is able to dictate the crystalline phase of the whole system.

### Photolithographic microprocessing for restructured phase-stable CsPbI_3_ perovskite films

We embed our PbI_2_-based interface into our thin films through controlled photo-thermal restructuring of the CsPbI_3_ and elucidate this process using a combination of in situ high-temperature grazing incidence wide-angle x-ray scattering (GIWAXS; Supplementary Fig. 11) and laser heating techniques (full details of the characterization approach are provided in the Supplementary Note 3). For the purely thermal process (Supplementary Figs. 12 and 13), the production of endogenous PbI_2_ begins near the yellow-to-black phase transition (320 °C) and continues for higher temperatures. Using focused 532 nm laser light which has an energy below the absorption edge of δ-CsPbI_3_ (Supplementary Fig. 14), prolonged exposure similarly heats the material and locally introduces chemical changes that can be resolved using Raman spectroscopy (Supplementary Fig. 15). In order of increasing radiation exposure, the laser-induced structural evolution is readily indexed via its unique Raman scattering signatures (Supplementary Fig. 16), where first endogenous deposits of PbI_2_ are formed and, at relatively high exposures, the material is ablated (Supplementary Fig. 17) to form PbO_2_. Notably, the intentional introduction of excess PbI_2_ has become routine for passivating deep traps in lead triiodide thin film devices^19^, rather than creating them. In relatively small, localized quantities (i.e. not permeating throughout the grain boundaries or pin holes of the polycrystalline film) the introduction of a well-defined PbI_2_ interface is expected to be relatively benign and not impact the optoelectronic properties of the perovskite film^19,20^.

**Fig. 2:**
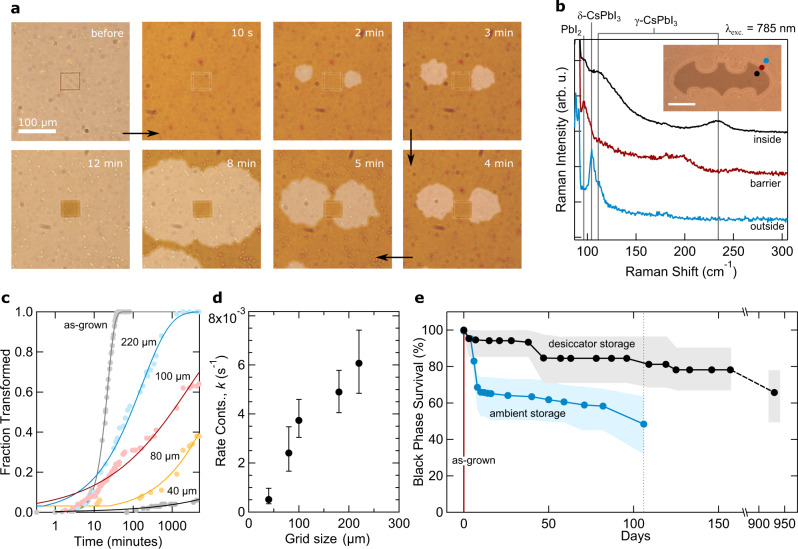
Photolithographic patterning and long-term stability of microprocessed films. **a** Optical imaging of a microprocessed 50 μm square before and after thermally quenching, stored under an ambient atmosphere (42%RH). The darker regions indicate the black phase, while the growing lighter parts are areas of the film that have transitioned to the yellow non-perovskite phase. **b** Micro-Raman characterization of a phase-stable microprocessed area; micro-Raman spectra are recorded inside (black), outside (blue) and on the border (red) of the microprocessed area, with an optical image of this area and corresponding measurement points (circles) inset. The image scale bar represents 50 μm. The labeled vertical lines link the detected modes to their assigned chemical species. **c** Temporal stability of black thin films stored under ambient conditions (42–48%RH) and processed with the different grid sizes indicated. The trends (solid lines) are modeled using the Johnson-Mehl-Avrami-Kolmogorov (JMAK) model, through Eq. (1). **d** The average transformation rate constant, *k*, over five samples as a function of microgrid size, as derived from Avrami plots (see Supplementary Fig. 28). The error bars correspond to the upper and lower bounds of determined values. **e** Comparison of the long-term phase stability of an as-grown film (red) stored in a glovebox with films installed with a 40 μm grid and stored under dry (black) and ambient, unregulated (blue) environments. Circle markers indicate the average fraction of surviving black-phase squares of five samples each with their upper and lower limits depicted by the solid fill region. The blue broken vertical line indicates where the ambient study was stopped due to moisture damage (Supplementary Fig. 31).

Next, we laser process micrometer-scale patterns into as-grown δ-CsPbI_3_ thin films (Supplementary Fig. 18) as a strategy for interface-driven stabilization within the enclosed area of the perovskite film; the full details of the approach can be found in the Supplementary Note 4. For our roughly 200 nm thick CsPbI_3_ films, the degree of structural ablation on the thin film surface is evaluated by height line scans taken across micro-processed tracks and, by regulating the laser power, photolithographic patterns are formed using a 458 nm laser power of 300 W/cm^2^ (with a writing at 10 mm/s) without significantly oxidizing/ablating the film surface (Supplementary Fig. 19). To elucidate the stabilizing effect of a micro-pattern at a local level, we fabricated an array of isolated 50 μm-squares across a film and recorded the retention of black film within the pattern borders (Supplementary Fig. 20). Figure 2a displays a single fabricated 50 μm-square shortly after thermal quenching; within minutes, yellow phase nuclei appear and spread toward the patterned border and are stopped from propagating further by the barrier. Beyond 10 minutes, the interior is rendered an ambient-stable black phase while the non-patterned film turns yellow.

Beyond simple shapes, arbitrary photolithographic patterns also prove effective at stabilizing a black interior film following annealing (see the inset of Fig. 2b). Comparing to reference Raman scattering data (see Supplementary Figs. 21, 22, 23, and 24), the micro-Raman spectroscopy contained in Fig. 2b confirms the formation of an inner ambient-stable perovskite phase, enclosed by a PbI_2_-based barrier that borders an outer δ-phase film. The introduction of PbI_2_-like signals are further verified using GIWAXS (Supplementary Fig. 25), with the micro-Raman signal recorded from the micropatterned border resembling more an amorphous-PbI_2_ system^21^, rather than a crystalline one (see the Raman data catalog shown in Supplementary Fig. 16).

### Phase energetic mechanism of microgrid

Developing a technologically relevant solution, the impact of the laser processing footprint (~4 μm) on the final film must be considered. Using a 0.4NA objective to write, the upper limit of the footprint is ~4 μm, i.e. the width of the embedded tracks directly measured via linescans (Supplementary Fig. 19). Thus, the ratio of the remaining photo-active surface area to the microprocessed footprint (Supplementary Fig. 26) remains relatively high until the length of the grid squares approaches the same order of magnitude as the footprint. We subsequently chose to limit the smallest size of the microgrid to 40 μm, retaining about 75% of the optically active perovskite film area. We implement small-scale, tessellating grid patterns of varying sizes and examine the phase transform kinetics under an ambient atmosphere (example raw data and analytical approach are contained in Supplementary Note 5). Tracking the decay of black squares over time (Supplementary Fig. 27), we find that employing smaller grids renders the perovskite films more stable. For a constant temperature (*T*) and RH, the phase transformation kinetics (Fig. 2c) are modelled using the Johnson-Mehl-Avrami-Kolmogorov (JMAK) equation which describes the fraction of transformed perovskite, *f*, over time (*t*) via:

1 $$f(t)=1-{\exp }\left(-k.{t}^{n}\right)$$

Here *n* is a constant and *k*(T) is the thermally activated rate constant which is given by the product of the number of transformation attempts at a molecular level (*k*_0_) and the activation factor that depends on the activation energy (*E*_*b*_) and the thermal energy (*k*_*B*_*T*; where *k*_*B*_ is Boltzmann’s constant), through $k\left(T\right)={k}_{0}{{\exp }}\left({-E}_{b}/{k}_{B}T\right).$ In this form, *k* absorbs both the phase nucleation and growth terms, with nucleation being sensitive to humidity in our case^8^. Constants *n* and *k* are determined by transforming Eq. (1) to yield a straight line with slope *n* and intercept ln(*K*) via:

2 $${{{{{\rm{ln}}}}}}\left(-{{{{{\rm{ln}}}}}}\left[1-f\left(t\right)\right]\right)={{{{{\rm{ln}}}}}}\left(k\right)+n{{{{{\rm{ln}}}}}}(t)$$

Evaluating the phase transformation kinetics as a function of grid size, their corresponding Avrami plots (Supplementary Fig. 28) reveal two linear regimes during their transformations. These arise via two different mechanisms and at two different length scales across the stabilizing microgrid. Specifically, the 3D structure of the microgrid reinforces interfacial contact throughout the film, extending the nano-meter scale in which the perovskite phase can interact and be anchored. This factor directly inhibits nucleation formation at the local level, i.e. within a single microgrid area. Conversely, at longer length (and time) scales, the microgrid partitions the film into structurally isolated domains, limiting the potential damage of each nucleation to a single grid area. These two factors govern the different regimes identified in the Avrami plots (Supplementary Fig. 28), and work together to enable long-term stability. For instance, in regime I, yellow phase nuclei seed and spread, and follow the evolution of the as-grown thin film. In regime II, further expansion of the yellow phase is prevented and the reaction becomes nucleation limited.

We subsequently determine the reaction rate constants over regime II as a function of the grid size and present the findings in Fig. 2d. Given that the Avrami exponent (*n*) also declines with smaller grid sizes (Supplementary Fig. 28), the interdependence of *k* and *n* complicates a direct comparison of the transformation rate values calculated^22^. Although, a reduction in *n* does agree with our ab initio calculations and suggests that the transformation mechanism itself is interrupted and, importantly, suppressed.

The stabilizing effect of smaller microgrids infers that *E*_*b*_ increases (Fig. 2d), however, the exact functional relating the transformation barrier height to the grid size is unclear (it will depend on factors like intra-granular transmission and film thickness). Additional non-local phase behavior is captured using in situ optical microscopy of different size grids after cooling from the high-temperature perovskites phase and storing the microgrid film in an ambient environment (see Supplementary Fig. 29). In particular, portions of the polycrystalline film outside of the enclosed microgridded area are also observed to have their phase decay rate slowed. Shortly after returning the CsPbI_3_ film to room temperature, micropatterning is seen to interrupt normal phase decay and delays the yellow phase transition for regions of the film connected through a continuous spread of black phase, up to millimeters away from the microgrid outer walls. This distance encompasses hundreds of individual grains in the film; structural coherence at this scale is impossible, making their coordination intriguing (Supplementary Fig. 29).

Black-to-yellow-phase formation imposes a ~7% drop in the crystal volume^23^ (per formula unit) and a reorganization of the atomic structure at the perovskite/substrate interface^16^. Like dominos, the abrupt change in nanograin volume and structure explains the lateral knock-on effect observed between grains at the thin film-level^24,25^. Conversely, neighboring black grains will pack to form an ensemble experiencing long-range interfacial forces^26^ which help by elevating the stability of all connected perovskite grains. Embedding a 3D microgrid is suggested to strengthen this collective phase phenomenon by introducing a periodic anchoring structure on a functional length scale, i.e., tens of microns in this case.

### Long-term stability and embedded interfacial microstructure

Keeping with small-scale 40 µm grid patterns, the long-term phase stability of microprocessed perovskite thin films is evaluated over the course of several months to years. While the half-life ambient stability of as-grown films is roughly 10 minutes (Supplementary Fig. 3), the introduction of a microgrid (Supplementary Fig. 30) extends this to over 100 days (Fig. 2e). The enhanced stability exhibited here within the microprocessed area is substantial compared to substrate strain-induced stabilization alone^16^ (Supplementary Fig. 2) and is directly attributable to the microgrid. Notably, after the saturation of yellow-phase nucleation sites (which is accelerated by ambient water^8^) and an initial decrease in black phase (regime I), the majority of perovskite grains near the center of the squares survive a moisture-rich environment during this time (regime II). This highlights the non-local nature of the embedded interface, which boosts moisture resistance many tens of microns across the thin film. Notably, the stability test was eventually stopped due to observation of widespread water damage across the film (Supplementary Fig. 31).

Long-term phase stability experiments were also conducted (Supplementary Fig. 32) on equivalent microprocessed thin films shielded from ambient moisture (Fig. 2e); after an initial drop of ~4% in the first 5 days, processed films retain up to ~95% of the black phase at 120 days and up to ~75% beyond 2.5 years. This enhancement in stability is directly attributable to the shifted phase energetics within the enclosed microgrid and the lattice anchoring mechanism prescribed above. Additional experiments revealed that important parameters like the choice of substrate (Supplementary Fig. 33) and the thin film thickness (Supplementary Fig. 34) did not significantly diminish the stabilizing effect of the microgrid. Furthermore, thermal- and photo-stability tests (Supplementary Fig. 35) of the processed films showed good resistance to these important stresses over the course of a few days.

**Fig. 3:**
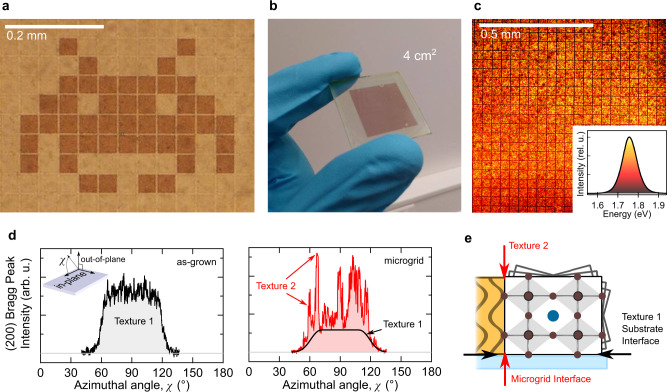
Scaling photolithography and probing of microgrid interface. **a** Image of graphic encoded into a microgrid thin film using selective laser destabilization. **b** Photograph of an ambient-stable microgrid (40 µm) thin film after annealing. **c** Confocal fluorescence micrograph of a processed CsPbI_3_ perovskite thin film (488 nm excitation/695–705 nm detection). A representative full PL spectrum is shown in the inset and acts as the color scale of the fluorescence image. **d** GIWAXS intensity of (200) γ-CsPbI_3_ scattering planes measured with and without a microgrid, as a function of azimuthal angle (*χ*) in the ***q***_*z*_ vs ***q***_*x,y*_ image of reciprocal space. For simplicity, the missing wedge is overlooked (Supplementary Fig. 39) to better resolve the unique contributions arising from Texture 2, i.e. the inset illustrates the angle *χ* relative to the 2D x-ray detector. **e** Illustrations of the anisotropic origins of Textures 1 and 2.

In contrast to as-grown films, which are vulnerable to the spread of a single yellow-phase nucleation event, the restricted connectivity across the gridded polycrystalline network limits the impact of each nucleus and is a key contributor toward realizing long-term stable devices. To explicate this feature, laser-triggered nucleation is used to induce yellow phase formation in selected squares of a stable perovskite microgrid (Supplementary Fig. 36). Spreading out from an initial nucleation site, the growing yellow phase reaches its first border wall near 1 minute and is prevented from propagating further. This confirms the absence of physical continuity between neighboring domains, which effectively blocks structural crosstalk. Fig 3a shows how selective laser-nucleation is used to control the phase of targeted perovskites squares, to generate a pixelated image.

Increasing the size of the patterned area easily scales the process to stabilize whole films (Fig. 3b). The optical properties of the stable perovskite surface are found to remain intact across the processed thin film (Fig. 3c and Supplementary Fig. 37) and a common γ-CsPbI_3_ polycrystalline structure is preserved and no additional strain^16^ is introduced (Supplementary Fig. 38). However, 2D x-ray scattering images reveal the subtle restructuring of the perovskite crystal when it interfaces with the embedded grid (Supplementary Fig. 39). The normal polycrystalline thin film texture^26^ (i.e. the direction and distribution of oriented grains in the film; Supplementary Fig. 40 and 41) is altered by the microgrid, confirming additional anisotropy in the system. Taking the (200) peak as an example to make the point, Fig. 3d shows how the smooth distribution of the as-grown film (Texture 1) departs when the microgrid is introduced (Texture 2), appearing more irregular, although always superimposing and amplifying regions of Texture 1. These hot spots confirm the presence of a 3D interface throughout the grid (Fig. 3e). Considering the relative intensity of the two texture components, a quantitative analysis (Supplementary Fig. 42) decouples the relative scattering volume from which they originate. We find that at least one third of the film grains are influenced by the anisotropy imposed by the microgrid and, assuming grains bordering the microgrid are most influenced, this suggests grains located far away from the boundary are also driven to express Texture 2. The embedded interface realized via the photolithography is the origin of the added anisotropy, leading directly to the strong stabilizing effect outlined above.

### Thin film devices stabilized using a microgrid

**Fig. 4:**
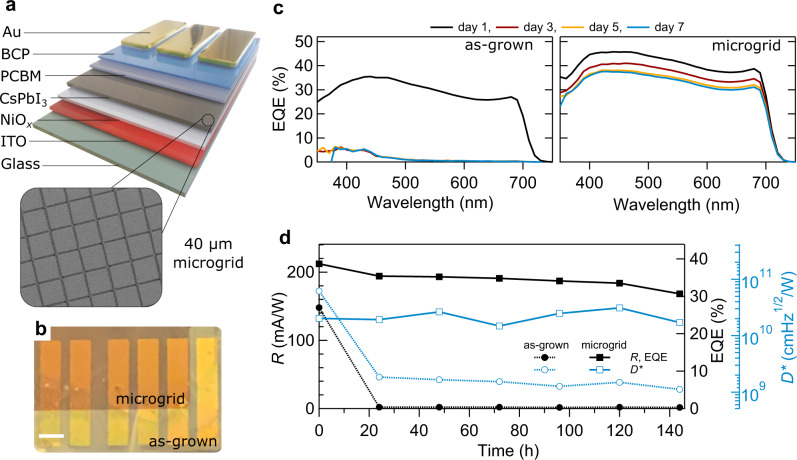
An embedded microgrid stabilizes CsPbI_3_ perovskite photodetectors. **a** Schematic illustration of device architecture with a scanning electron microscopy (SEM) image of the active perovskite layer with an embedded 40 μm microgrid. This inverted p-i-n type device architecture was chosen with indium tin oxide(ITO)/NiO_*x*_ acting as the anode and the phenyl-C 61-butyric acid methyl ester/bathocuproine (PCBM/BCP) layers acting as the cathode. **b** photograph (after first 24 h of ambient storage; scale bar represents 3 mm) of unencapsulated CsPbI_3_ photodetector devices stabilized by integrating a microgrid into a selected portion of the photo-active perovskite layer. **c** Tracking of effective quantum efficiency (EQE) spectra over 7 days for devices fabricated with and without the microgrid. **d** Figures of merit (operating at 690 nm and −0.5 V): EQE, responsivity (*R*) and specific detectivity (D*) of devices operating in an ambient environment (45–50% RH) for one week.

Thermal processing of the CsPbI_3_ perovskite layer (>320 °C) imposes limits on the choice of hole transport layers which can be used in optoelectronic devices based on this material, motivating the use of thermally robust inorganic oxide compounds, like NiO_x_. As a conceptual demonstrator, our microgrid technique is compatible with planar heterojunction devices and we find stabilizing otherwise-unstable CsPbI_3_ thin film devices is realized by simply laser patterning after spin coating the CsPbI_3_ layer and before annealing, keeping all other processing steps the same (details of the device fabrication are provided in the Supplementary Note 8). Figure 4a and b depict how this approach is translated into working, ambient stable CsPbI_3_-based perovskite photodetectors, with a comparison of device metrics across multiple batches of films shown in Supplementary Table 1. Once integrated, the endogenous, all-inorganic nature of the microgrid imposes no added restrictions on device fabrication. The performance of unencapsulated photodetector devices fabricated with and without a microgrid was tracked for one week (Supplementary Fig. 43). Devices based on as-grown films exhibit rapid phase decay within the first day of ambient operation; in this time, the external quantum efficiency (EQE) spectrum shifts from the broad, characteristic response of the black CsPbI_3_ perovskite phase to a narrow response, indicative of the large bandgap yellow-phase (Fig. 4c). In comparison, the EQE spectrum recorded from devices fitted with a microgrid remains relatively stable, translating into stable figures of merit (Fig. 4d) and the device yields relatively fast response time under pulsed excitation (rise/fall times of ~2 μs; Supplementary Fig. 44). Additional device characterization was carried out under different light intensities and varied external bias (Supplementary Fig. 45, 46 and 47). We further demonstrate that this stabilization approach is compatible with mixed halide perovskite devices (Supplementary Fig. 48) and that shielding CsPbI_3_ photodetectors from ambient moisture (Supplementary Fig. 49) further enhances their operational stability. The latter is considered analogous to shielding the device from moisture via encapsulation.

## Discussion

Next we contextualize our stabilization approach and device performance results (Fig. 4) with respect to the current state of the art. First, the use of a stabilizing microgrid allows us to retain the bulk properties of CsPbI_3_ which are desirable for efficient optoelectronic devices, i.e., an unchanged bandgap energy and intrinsic, bulk carrier transport. This is a key distinction when comparing our approach to those reported previously. For example, common scalable stabilization methods^27^ like reducing the crystal dimensionality (nanocrystals/quantum dots), altering the composition, or capping the crystal surface^11^ (i.e. ligands, PMMA, etc.), inherently disrupt the bulk optoelectronic properties of CsPbI_3_ perovskite in the film. Subsequently, considering the vast processing options available^28^, the figures of merit of different APbI_3_-based photodetector architectures vary significantly^29^. This is because certain design choices will influence aspects of performance differently, i.e. the use of thicker active perovskite layers will increase the responsivity at the expense of limiting the temporal response (rise/fall time). As such, tradeoffs must be made when designing the intended function.

While the responsivity of our photodetector devices (R) are only modest compared to current perovskite-based designs and performance, once the capability of detecting low-level light signals is considered, the specific directivity (D*) becomes comparable to modern perovskite-based devices^29^. Importantly, our devices exhibit a fast temporal response (rise and fall times of ~2 μs), which nears the state-of-the-art response times for APbI_3_-based devices^30^. We ascribe this to the preservation of bulk carrier transport^31^ and relatively thin film thickness (~200 nm) within our devices. On the other hand, the operational stability of our unencapsulated and pure CsPbI_3_-based devices in Fig. 4d is relatively stable (T_80_ ~150 h). There are a limited number of reports outlining unencapsulated CsPbI_3_ devices which do not possess some form of crystal surface modification (e.g. functional ligands or PMMA crystal capping), to shield them from the effects of ambient moisture. Within this context, the stability of our unencapsulated CsPbI_3_ perovskite devices fashioned using a generic solution processing approach is particularly novel^28^. Notably, once moisture is shielded and/or the phase is reinforced through mild halide mixing (CsPbI_2.75_Br_0.25_; see Supplementary Fig. 48), we find no detectable drop in device performance over 7 days of testing. With respect to the other stabilization approaches used to arrive at functional forms of black CsPbI_3_ thin films^27^, our strategy is expected to be entirely orthogonal to current options. Further, we anticipate that they can be effectively combined for superior long-term material stability and device operation.

In conclusion, we have presented an effective strategy for embedding an interfacial microstructure (a PbI_2_ microgrid) into stable CsPbI_3_ perovskite thin films and devices, using a coarse photolithographic approach. The microgrid is shown to dramatically increase the long-term stability of black CsPbI_3_ thin films (beyond 2.5 years in a dry environment) by increasing the phase transition energy barrier (*E*_b_) and limiting the spread of potential yellow phase formation to a single, isolated domain of the grid. Using stabilized photodetectors, integration of a microgrid into normally unstable planar CsPbI_3_ perovskite devices is shown to be a simple and effective strategy toward stable ambient operation.

## Methods

### Materials preparation

Perovskite thin films: Cesium iodide (CsI, 99.9%) was purchased from Fisher Scientific. Lead iodide (PbI_2_, 99.9%), lead bromide (PbBr_2_, 99.9%), anhydrous dimethyl sulfoxide (DMSO) and gamma-Butyrolactone (GBL) were purchased from Sigma-Aldrich. The perovskite precursor powders were weighed and treated at 80 °C under vacuum in a vacuum oven for 2 h to remove any trapped moisture before use. Phenyl-C61-butyric acid methyl ester (PCBM, 99%) and Bathocuproine (BCP, 99%) were purchased from TCI chemicals and used as received. The perovskite precursor solutions were prepared by dissolving equimolar ratios of CsI and PbI_2_ in a DMSO:GBL (4:1, v-v) solvent mixture to result in precursor solutions with molar concentration ranging from 0.8 M to 1.5 M. In case of the Br doped perovskite, the ratio of PbI_2_/PbBr_2_ was varied accordingly. The precursor solutions were then stirred at 60 °C for 12 hrs. PCBM was dissolved in 1,2-dichlorobenzene (99%) at 25 mg/ml concentration and BCP was dissolved in methanol (99.9%) at 2 mg/ml concentration and stirred at 60 °C for 4 hrs. All the solutions were filtered using a 0.2 µm PTFE (polytetrafluoroethylene) filter before use. Thin films were spin-coated in a nitrogen glove box on targeted substrates at 1500 rpm for 30 s and 3000 rpm for 60 s. The film was then transferred onto a hotplate for annealing at 160 °C for 60 s. The resultant film thickness varied between approximately 150 to 350 nm, depending on the concentration of the precursor.

Bulk CsPbI_3_ materials were similarly synthesized, whereby crystallization took place in a static environment on glass substrates (i.e. drop cast).

Thermally quenched samples were placed on a hotplate under nitrogen gas flow for 1 minute to fully transition the CsPbI_3_ material black, then removed and placed immediately onto a RT piece of metal, to rapidly cool it. The materials were exposed to an ambient atmosphere during the cooling and subsequent study, unless specified otherwise.

CsPbI_3_ thin-film thickness series: For the thickness series, 0.8 M, 1 M, 1.2 M & 1.5 M CsPbI_3_ precursor solutions were spin-coated on ITO coated glass substrates to result in 150 nm, 220 nm, 280 nm & 330 nm thick CsPbI_3_ films, respectively, under same spin conditions described above. The substrates were then transferred to the home-built laser patterning system and 40 µm microgrids were patterned (over 2 × 2 cm^2^ area) on the perovskite layer as described elsewhere in the manuscript (RH ~ 40%). The substrates were then annealed at 320 °C for 2 mins to transform from the yellow to black phase. The substrates were then stored under ambient conditions (RH ~ 40%) for weeks and photographed every day.

Amorphous PbI_2_: Amorphous lead iodide (a-PbI_2_) films were spin-coated under nitrogen atmosphere (glovebox) from a 1 M solution of PbI_2_ in N,N-dimethylformamide (DMF) and dried at 70 °C for 1 min.

Photodetector Fabrication: Glass substrates with patterned indium tin oxide (ITO) layer purchased from Naranjo B.V. were sequentially washed with soap (2% Hellmanex III), deionized water, acetone and isopropanol in an ultrasonic bath for 30 mins each step and dried in an oven. The substrates were treated with UV-ozone for 60 minutes prior to film deposition. The ITO substrates were then sputtered with a metallic Ni layer using a Balzers BAE 370 sputter coater and annealed at 300 °C for 1 h to result in a ~25 nm NiO_x_ layer. The NiO_x_ layer was then treated with UV-ozone for 30 minutes prior to perovskite deposition. Afterwards, the perovskite precursors were spin-coated at 4000 rpm for 60 s and left undisturbed (RH 25 %) for 1 h to promote uniform crystal growth as described elsewhere^32^. The films were then annealed at 160 °C for 2 minutes to complete the film formation. For the devices, a 1 M concentration CsPbI_3_ or CsPbI_2.75_Br_0.25_ precursor solution was used to result in ~220 nm CsPbI_3_ or CsPbI_2.75_Br_0.25_ film, respectively. The substrates were then transferred to the home-built laser patterning system and 40 µm^2^ microgrids were patterned on the perovskite layer as described elsewhere in the manuscript (RH ~ 40%). The substrates were then annealed at 320 °C for 2 mins to transform from the yellow to black (α) phase. The PCBM layer was then spin-coated at 1000 rpm and annealed at 100 °C for 30 mins to result in a ~25 nm film. The BCP layer was then spin-coated at 4000 rpm and annealed at 100 °C for 2 mins to result in <5 nm film. Finally, the substrates were transferred into a Balzers thermal evaporator where the gold (100 nm) cathode was deposited through a shadow mask at a vacuum pressure of <3 × 10^-5^ mbar. The active area of the devices was 6 mm × 2.5 mm. The substrates were then stored under ambient conditions for the rest of the measurements unless indicated otherwise.

### Characterizations

Photodetector Characterization: Dark-current and photo-current under white light were measured using a Keithley 2602 A Source-Measure unit and a Abet solar simulator under 100 mW/cm^2^ (1 sun) simulated AM 1.5 G illumination. The scan was between −0.5 V to 0.5 V at a step rate of 1 V/s and a step size of 0.01 V.

EQE measurements were performed using a Bentham PVE300 spectral response system, with a Xenon/Quartz halogen lamp and a Bentham TMc300 single monochromator with a step size of 10 nm. Measurements were calibrated using a Si photodiode. Responsivity is calculated from the EQE values, as in equation:

3 $$R=\frac{e}{{hc}/\lambda }\times{{{{{\rm{EQE}}}}}}$$

where *e* is the electron charge, *h* is Planck’s constant, *c* is the speed of light, and *λ* is the incoming wavelength.

Specific detectivity, the ability of a device to detect weak signals in the presence of noise and is determined by the device responsivity and the noise current, or noise equivalent power (NEP), as D* = √(AΔf)/NEP. Assuming that shot noise is the main contributor to the NEP in dark conditions, and that the contributions of Johnson and flick noise are insignificant, D* is defined as:

4 $${D}^{*}=R/{(2e{J}_{{dark}})}^{1/2}\,({{{{{\rm{cm}}}}}}{{{\mbox{Hz}}}}^{1/2}{{{\mbox{W}}}}^{-1})$$

where *J*_*dark*_ is the dark current density of the device. EQE values are obtained for a wavelength range from 350 to 750 nm, and EQE, *R* and D* tracking are plotted for a wavelength of 680 nm. In both cases, the devices were biased at –0.5 V. The measurements were performed under ambient conditions at a RH level between 45–50%, unless indicated otherwise.

A PAIOS Fluxim system with LED was used to measure the rise and decay time and the capacitance of the devices. The pulsed J-V characteristics were measured from 2 V to −1 V with 50 ms, 1 ms, and 25 ms as the pulse length, rise time, and measurement time, respectively.

The measurements were averaged over 3 runs with a sweep offset voltage ranging from 0 V to −1 V in steps of −0.1 V.

Scanning Electron Microscopy (SEM): SEM characterizations on the samples were carried out with a FEI Quanta FEG-250 SEM. An acceleration voltage of 2 or 5 kV was applied during the measurements to reduce charging on the sample surface.

Synchrotron-based Grazing Incidence Wide Angle X-ray Scattering (GIWAXS): The GIWAXS data were collected at two different synchrotron light sources, using comparable wavelengths.

European Synchrotron Radiation Facility (SERF): The in situ GIWAXS data were collected at BM01 (SNBL/ESRF in Grenoble, France) using the PILATUS@SNBL diffractometer^33^. The monochromatic beam (*λ* = 0.95774 Å) and the parameters of the detector were calibrated on LaB_6_ powder using PyFAI^34^. The obtained calibrations were implemented to Bubble for further azimuthal integration of 2D images. The resulting unit cell models were refined using the Le Bail method in Fullprof^35^. The sample was positioned parallel to the synchrotron beam, and optimal angle with a pronounced diffraction pattern was fixed for further variable temperature measurements. The temperature on the sample was changed using the nitrogen hot blower, calibrated using the dependence of silver unit cell parameters, and preliminarily measured at the sample position.

ALBA Synchrotron: Room temperature GIWAXS of CsPbI_3_ thin films were recorded at NCD-SWEET beamline (ALBA synchrotron in Cerdanyola del Vallès, Spain) with a monochromatic (λ = 0.95764 Å) X-ray beam of 80 × 30 µm^2^ [H × V], using a Si (111) channel cut monochromator. The scattered signal was recorded using a Rayonix LX255-HS area detector placed at 241.1 mm from the sample position. The reciprocal *q*-space and sample-to-detector distance were calculated using Cr_2_O_3_ as calibrant. An incident angle (*α*_*i*_) of 1° was chosen to ensure full penetration of the X-ray beam through the layer. Continuous N_2_ flow over the sample was employed during the measurements. Collected 2D images were azimuthally integrated using PyFAI^34^.

Raman scattering experiments: Raman spectra recorded using 532 nm excitation (laser power 15 mW) were acquired on a Labram-HR Evolution intrument from Jobin-Yvon-Horiba company. A neutral density (ND) filter wheel was used to control the indicant power and the laser light was focused using a ×40 long working distance (LWD) objective. At 785 nm, XPlora (from the Jobin-Yvon-Horiba company) spectrometer with a the laser power of 0.4 mW is used with an LWD objective of ×50. For high-temperature measurements, the CsPbI_3_ material inside a heating stage (HFS600, Linkam) is connected to a temperature controller. The sample was heated between 25 °C and 330 °C with spectra recorded at different stages of the ramp. For each measurement, an exposure time of 1 minute was used.

DFT calcuations of Raman spectra: The second-order atomic force constants and associated phonon eigenvectors and eigenvalues were obtained from first-principles lattice dynamics using the crystal structures and technical setup reported elsewhere^36^. The total energy and forces were computed for both the yellow non-perovskite δ-CsPbI_3_ and the black γ-CsPbI_3_ perovskite phases, using density functional theory (DFT) via the PBEsol functional in VASP^37^ and with the phonons processed using Phonopy^38^. The k-points are converged at 8 × 8 × 8 and 8 × 10 × 8 with Monkhorst-Pack grid for the γ-phase and δ-phase, respectively. The converged cut-off energy is 600 eV and the total energy convergence threshold is 1 × 10^−6^ eV. The Raman intensities were calculated using the SpectroscoPy toolkit available from https://github.com/JMSkelton/Phonopy-Spectroscopy. Note that for a perovskite cubic perovskite, no first-order Raman is expected. Due to dynamic structural fluctuations, it is often observed in practice as the local structure in fact is closer to the orthorhombic phase with strong local octahedral tilting and atomic displacements from the cubic parent structure.

Photoluminescence (PL) microscopy: PL spectral mapping over the sample was carried out on a home-built inverted optical microscope (Ti–U, Nikon) in a confocal mode. An air objective of 0.92 NA and 60× magnification was used. A 532 nm diode laser was used as the excitation light source. The excitation power density was regulated with a set of neutral density filters. A pair of half- and quarter-wave plates was used for converting the linear-polarized laser light into circular-polarized for excitation. A 545 nm long-pass filter was used in front of the entrance of the spectrograph. A piezostage was used for sample scanning.

Confocal Fluorescence Microscopy: Confocal fluorescence micrographs were acquired on an inverted epi-fluorescence confocal microscope (Olympus IX81), λ_exc_ = 488 nm, equipped with an air immersion objective lens (Olympus, 40×, 0.9 NA). Detection of fluorescence emission (λ = 780–800 nm) was done with a PMT and pixel dwell time of 20 μs. The images collected have fields of view of 158.72 × 158.72 μm^2^ with a pixel size of 248 × 248 nm^2^, which was zoomed in with a ×2 magnification. Multiple images were taken at several focus planes and processed using ImageJ to obtain the average projection of the fluorescence signal. Background intensity was corrected by thresholding using ImageJ to remove signal contributed by the background.

Optical Absorption: Diffuse reflectance spectra (DRS) were recorded in the wavelength range between 300 nm to 950 nm with a Perkin Elmer Lambda 950 UV-Vis spectrophotometer. As reference, BaSO_4_ powder was used. The diffused reflectance (*R*) was converted to function *F*(*R*), which is proportional to the rate between absorption and scattering, using the Kubelka-Munk function:

5 $$F\left(R\right)=\frac{{\left(1-R\right)}^{2}}{2R}$$

Emission lifetime imaging: Fluorescence lifetime microscopy was performed at room temperature using an inverted-type scanning confocal microscope (Leica TCS SP8 SMD) with a 10x air objective (Olympus), supported by PicoQuant Symphotime software. A 470 nm pulsed diode laser (PicoQuant PDL 800-B) was used for excitation with a repetition frequency of 20 MHz. The emission detection window was set at 650–750 nm.

The recorded emission lifetimes appear as a superposition of multiple radiative transitions. The decay of all emission signals presented in the main text are analysed within a framework of summing the exponential decays of multiple decaying components relative to the time of excitation (*t*_0_), with differing decay constants (*τ*_j_) and amplitudes (*A*_j_). For the PL lifetimes recorded in Figure S33 bi-exponential models yielded the best fit, using:

6 $$I\left(t\right)={A}_{1}{{\exp }}[\left(t-{t}_{0}\right)/{\tau} _{1}]+{A}_{2}{{\exp }}[\left(t-{t}_{0}\right)/{\tau}_{2}]$$

Optical microscopy imaging: Optical images of the microprocessed thin film surfaces were recorded using a desktop Leica DMS300 optical microscope.

Atomic Force Microscopy (AFM): AFM maps of 15 × 15 µm^2^ were recorded at a scan speed of 0.35 Hz with a pixel size of ∼33 × 33 nm. The thin film surface was characterized in tapping mode using OMCL-AC160TS-R3 probes (spring constant of ∼26 N/m) at a resonance frequency of 300 kHz (±100) using a Cypher ES system (Asylum Research) at 32 °C. The acquired AFM data were processed and analyzed using the open-source software package Gwyddion^39^.

Density functional theory (DFT) modeling of interface: The dynamic calculations performed for this report are all molecular dynamic (MD) simulations at 300 K or 600 K with a timestep of 2 fs and a total simulation time exceeding 160 ps. CP2K^40,41^ was used to calculate the forces and update the positions. The forces were calculated using density functional theory (DFT) with the PBE-D3(BJ)^42,43^ exchange-correlation (XC) functional. The combined Gaussian and Plane Wave (GPW) basis sets approach is used. The TZVP-MOLOPT-SR-GTH basis set and GTH pseudopotentials^44–46^ are used for all atoms. The cutoff energy is 400 Ry and the relative cutoff is 40 Ry. The calculations in which the lattice vectors were fixed were performed in the NVT ensemble at 300 K or 600 K, using a Nosé-Hoover thermostat^47–49^ with three thermostat beads and a time constant of 100 fs to control the temperature. For the calculations in which also the unit cells could move freely, the NPT ensemble was used by also applying an MTK barostat^50^ with a time constant of 500 fs to control the pressure at 1 atm. PLUMED^51^ was used to calculate the Pb-I-Pb bond angles during the MD simulation and to add the bias restraint to the I^−^ anions at the bottom of the slab.

## Supplementary information

Supplementary Information

## Data Availability

The authors declare that the data needed to evaluate the conclusions in this manuscript are present in the main text or the Supplementary Information. Experimental procedures, characterization of materials, computational details, Supplementary Tables and Supplementary Figures are available in the Supplementary Information. Additional raw data formats are available upon request to the corresponding authors.

## Code availability

The input, output, and processing scripts used to complete the materials simulations are available at https://zenodo.org/record/7341824#.Y3t3f1NKi0p. The commercial VASP code can be licensed from the University of Vienna (see the FAQ section on https://www.vasp.at/). The applied codes (Phonopy, CP2K and PLUMED) are freely accessible.
