# Supplementary material for: An embedded interfacial network stabilizes inorganic CsPbI3 perovskite thin films
Source: Nat Commun. 2022 Dec 6;13:7513. doi: 10.1038/s41467-022-35255-9 (PMC9727127; doi:10.1038/s41467-022-35255-9)
Supplement: Supplementary file 1 — Supplementary Information [file 41467_2022_35255_MOESM1_ESM.pdf]

Supplementary Information for:

An embedded interfacial network stabilizes inorganic $CsPbI_3$ perovskite thin films

Julian A. Steele*, Tom Braeckevelt, Vittal Prakasam, Giedrius Degutis, Haifeng Yuan, Handong Jin, Eduardo Solano, Pascal Puech, Shreya Basak, Maria Isabel Pintor-Monroy, Hans Van Gorp, Guillaume Fleury, Ruo Xi Yang, Vittal Prakasam, Zhenni Lin, Haowei Huang, Elke Debroye, Dmitry Chernyshov, Bin Chen, Mingyang Wei, Yi Hou, Robert Gehlhaar, Jan Genoe, Steven De Feyter, Sven M. J. Rogge, Aron Walsh, Edward H. Sargent, Peidong Yang, Johan Hofkens, Veronique Van Speybroeck*, and Maarten B. J. Roeffaers*

*Corresponding authors: julian.steele@kuleuven.be, veronique.vanspeybroeck@ugent.be, maarten.roeffaers@kuleuven.be

Inventory of Supplementary Information:

Supplementary Notes 1 to 8
Supplementary Figures 1 to 49
Supplementary Table 1
Supplementary References

Supplementary Note 1. Baseline stability of conventional solution-processed $CsPbI_3$ thin films

Materials under investigation: Both micron-scale, free-standing crystals (made using drop casting) and spin coated polycrystalline thin films of $CsPbI_3$ are fabricated (Fig. S1) and tested to reference the enhanced stability gained from fabricating thin films.

Baseline stability: We examine both bulk vs thin film stability shortly after thermal quenching under both ambient and nitrogen atmospheres (Fig. S2). The $CsPbI_3$ materials underwent a thermal quenching procedure and were placed under an optical microscope (Leica DMS300) for monitoring immediately after. A nitrogen atmosphere was introduced using a dispersive gas outlet which submerged the sample in free space.

After thermally quenching under both ambient and inert atmospheres (Fig. S2), free-standing bulk $CsPbI_3$ crystals turn yellow within tens of seconds under both environments. Thin films exposed to the same treatment last significantly longer due to the stabilizing effect of substrate clamping (*1*), taking roughly 40 minutes to fully transition yellow in an ambient atmosphere (42% RH) and over 10 hours when evaluated under nitrogen gas flow. Tracking the degree of black-to-yellow transformation with time (Fig. S3), we find that moisture accelerates phase decay in black $CsPbI_3$ thin films.

We aim to integrate our stabilizing technology within routine solution-processing methods; therefore, these data provide the baseline for which we stabilize the 200 nm-thick $CsPbI_3$ thin films using photolithographic patterning.

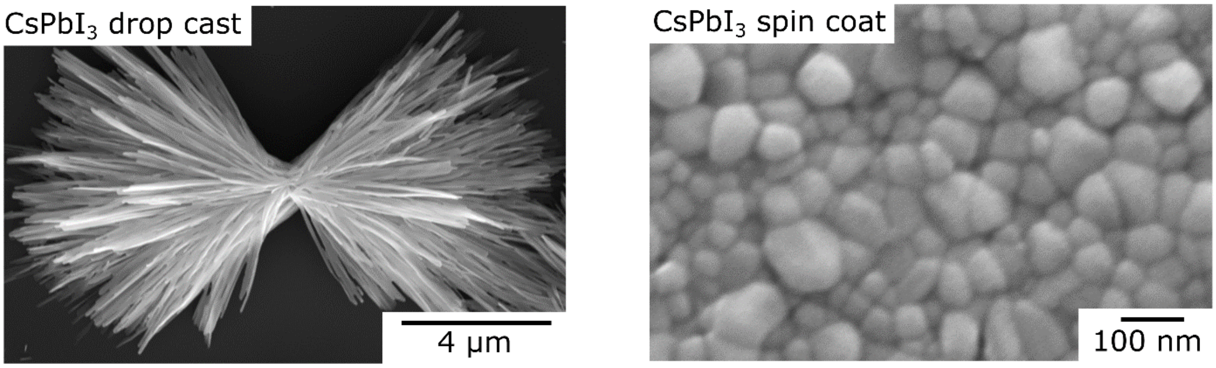

Fig. S1.

SEM images of $CsPbI_3$ samples prepared by spin coating (thin films and NCs) and drop cast depositions (powders). The thin films exhibit NCs ranging from roughly 50 – 200 nm, while the powders have a larger needle-like morphological arrangement, defined by the stacking of crystals which are relatively long (approximately ranging from 5 to 15 µm).

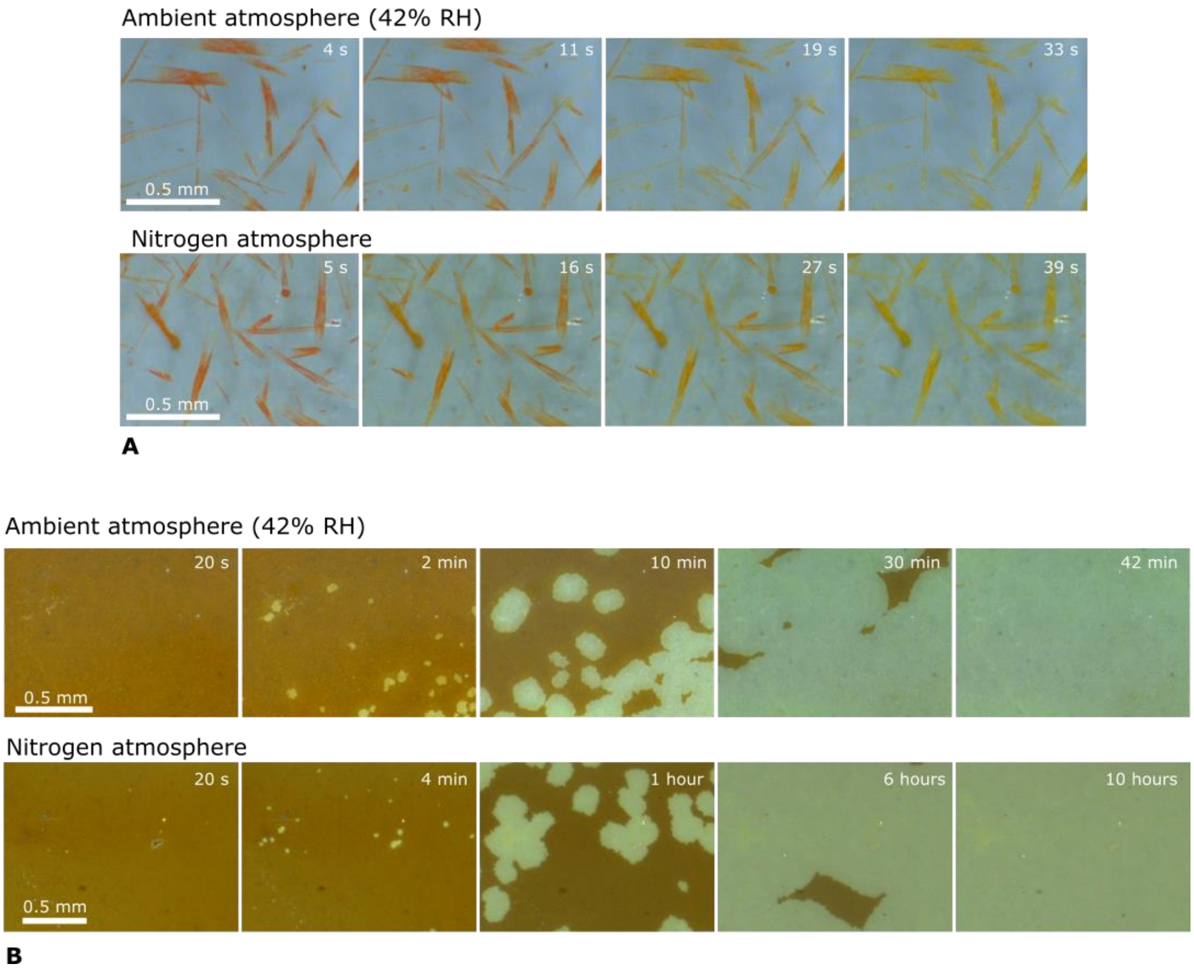

Fig. S2.

Optical images of $CsPbI_3$ materials after annealing at 330°C and thermally quenching to RT (22-24°C) to kinetically trap the black phase. (A) Bulk needle-like crystals and (B) as-grown thin films, in both a moisture-rich ambient atmosphere and immersed in a pure nitrogen gas flow.

Beyond the role of strain-induced stabilization in the thermally treated film, the relatively small grains in the thin film (50-200 nm) also has an additional stabilizing effect on the perovskite phase, due to a difference in the surface energy contributions of the competing γ-$CsPbI_3$ perovskite and δ-$CsPbI_3$ non-perovskite phases (*2*). This is, in part, important for the phase stability comparison carried out here, which constitutes a benchmark result to be improved on throughout this study.

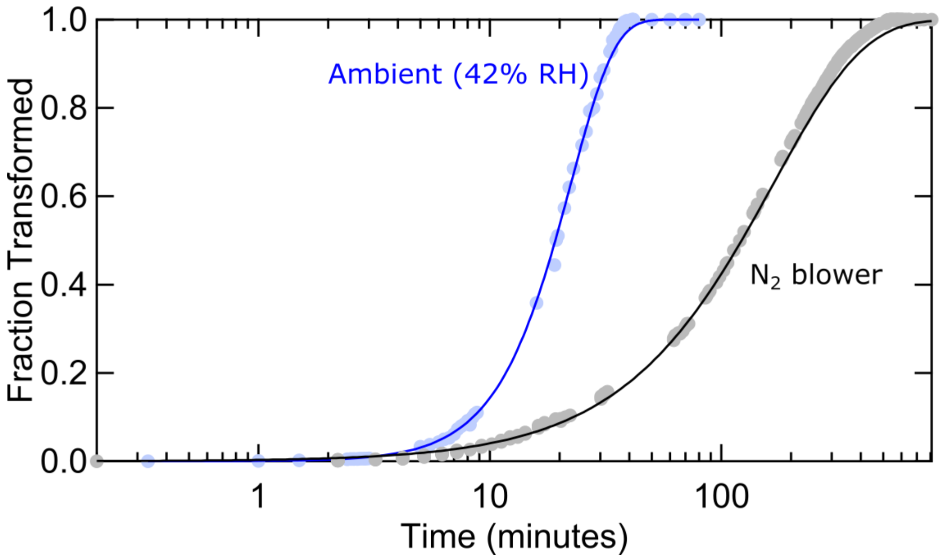

Fig. S3.

Temporal stability of black thin films stored under ambient (40-44%RH) and inert (nitrogen) atmosphere. These data were derived of a time-dependent image threshold analysis of the transformations imaged in Fig. S2. Fits to the experimental data (solid lines) are made using the standard JMAK model.

Supplementary Note 2. Simulations of surface octahedral restraint

MD simulations: Periodic simulation cells were used to track the Pb-I-Pb bond angles of the $CsPbI_3$ slab during the MD simulations, performed using CP2K (*3*), as shown in Fig. S4 for the four-layer model. The simulation cells were constructed from a 4x4x4 and 8x4x4 supercell of the conventional unit cell and a vacuum of 15 and 10 Å respectively to ensure that there are no periodic interactions in the z-direction. The lattice vector in the z-direction remained fixed for all calculations. To terminate the slab extra I^- anions are added to preserve the PbI octahedra. To maintain charge neutrality the same number of Cs^+ cations are added. The resultant relative dimension the simulated slab to the vacuum layer was 25 Å: 15 Å and 50 Å: 10 Å for the four- and eight-layer cells, respectively.

For each simulation, the reported bond angles are divided in 3 groups depending on their orientation with respect to the applied interface (at z=0), which is defined by the orientation of the Pb-I bonds in the initial α-phase (Fig. S4). Apart from using the slab model to study the effect of a variable constraint on the bond angle distribution (see Fig. 1C in the main text), we also investigated various other effects:

(I) The effect of fixing the cell vectors, and

(II) The effect of fixing the position of the bottom layer of I^- anions.

Details of approach (I): The effect of fixing the cell vectors, either at their equilibrium values at 300 K or 600 K, on the distribution of the bond angles is investigated using three independent simulations: one simulation with free cell vectors at 300 K (NPT ensemble), and two simulations with fixed cell vectors, one at 300 K and one at 600 K (NVT ensemble). For the MD simulation with free cell vectors at 300 K, there is a large difference between the bond angles oriented in the x and y directions (θ_x and θ_y), as shown in Fig. S6. This is the result of different octahedral tilting occurring in both directions at 300 K, as the rotation around the x-axis to go from the β-phase to the γ-phase, and effects θ_y and not θ_x.

In a second simulation, the lattice vectors were fixed to their average value at 300 K and this restraint partly inhibits the octahedral tilting observed in the free material, leading to larger bond angles oriented in the y and z directions and slightly smaller bond angles in the x direction. Thus, to further mimic the experimental lattice anchoring while strained due to a thermal expansion mismatch with the substrate, in a third simulation the lattice vectors are constrained to their value at 600 K. This is the corresponding temperature at which the perovskite structure is formed in the film and establishes an interface with the underlying substrate, which contracts much slower than the perovskite upon cooling, causing strain (*1*). In this case, Fig. S7 shows that the angles increase for both the strained in-plane directions (θ_x and θ_y) and for the perpendicular, out-of-plane direction (α_z), with respect to the 300 K situation in which the lattice vectors are free to move.

Details of approach (II): The I^- anions in the bottom layer of the four-layer slab were kept fixed in addition to fixing the lattice vectors in the x and y directions to their average value at 300 K. A harmonic restraining potential $V = Kd^2$ was applied to all I^- anions in one layer as shown in Fig. 1C of the main text, with K being the strength of the restraint and d the distance from their

initial positions. For $K = 0$, the I^- anions at the interface are free to move as normal. Fig. S8 shows the Pb-I-Pb bond angle distributions as obtained from this simulation, contrasting them to those values obtained in the prior MD simulation where the I^- anions are free to move but the lattice vectors are fixed on their 300 K values. These data further lead to varied bond angle energies in Fig. S9. One observes a large positive effect for the out-of-plane bond angles and a negligible effect for the in-plane bond angles due to the rotational freedom around the z-axis. The effect is the largest for the first layer and decreases for layers further from the restraint plane (Fig. S10B), giving a first indication of the range over which this restrain stabilizes the material.

Large eight-layer slab calculations: Due to the limited size of the four-layer slab, it is not optimal to examine how the interface effects translate through the $CsPbI_3$ layers from Fig. 1C in the main text alone. Therefore, those calculations were also performed for an eight-layer slab of $CsPbI_3$ in which the I^- anions at the bottom were restrained with a harmonic bias potential $V = Kd^2$, with $K = 1000$ kJ mol^{-1} Å^{-2} and d the distance between the initial and actual positions of the I^- anions (Fig. S10B).

Depth profile analysis of surface restraint: Considering layer-by-layer how the Pb-I-Pb angles are influenced beyond the anchored surface atoms (Fig. S10), a depth profile analysis suggests that the tilts are restricted only over a short range, vanishing after a few layers beyond the surface. Crystal surfaces generally contain the tail ends of structure-property distributions, including bonding angles, which facilitate nucleation sites for phase transitions.

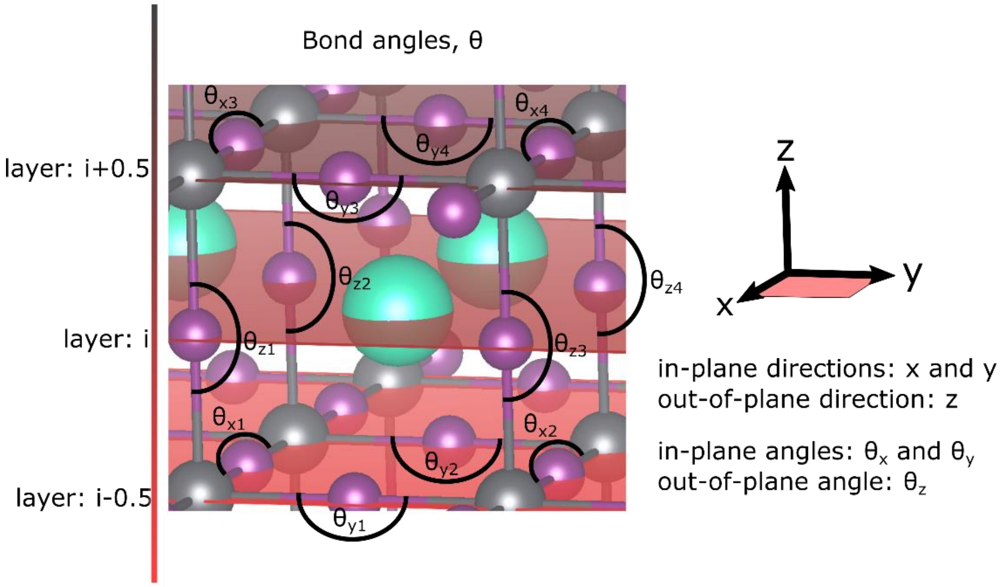

Fig. S4.

Enlarged depiction of the Pb-I-Pb bond angles of a larger 4 × 4 × 4 supercell of a slab with 4 layers of $CsPbI_3$, which are separated in 3 groups; θ_x, θ_y, and θ_z. These are defined relative to the in-plane (x and y) restrictions applied to the anions during the MS simulation, as outlined in Figure 1C of the main text. Cs^+ cations are cyan, Pb^{2+} cations are gray and I^- anions are purple.

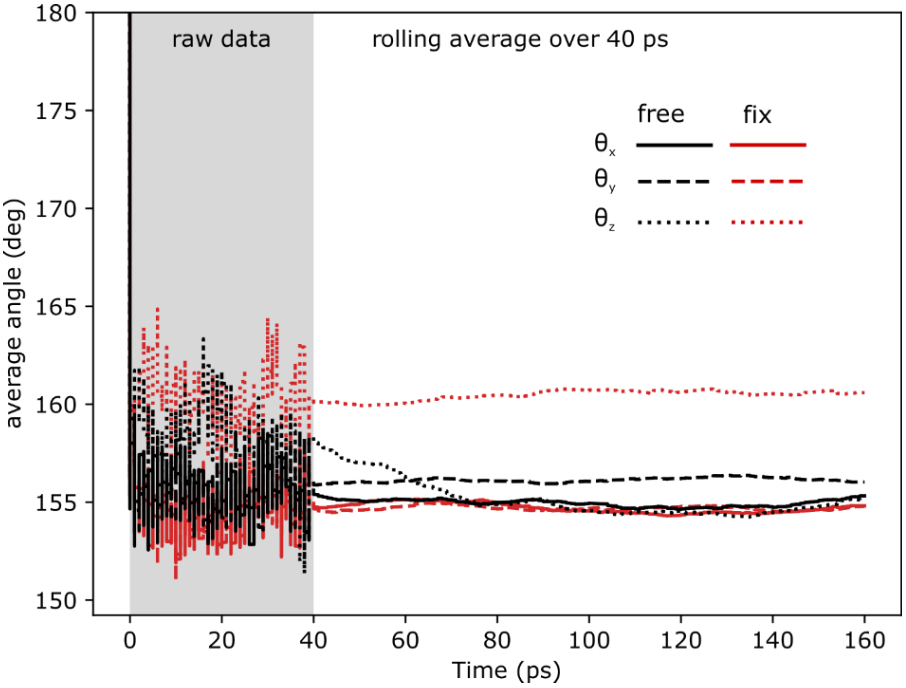

Fig. S5.

Pb-I-Pb bond angles oriented in the directions outlined in Figure S1. Raw data is presented early on in the simulation to show the rapid drop average bond angle when starting from an initial cubic structure (bond angle of 180) of the four-layer $CsPbI_3$ slab at 300 K. The black lines represent the results for the simulation where $CsPbI_3$ was free and the red traces represent the results where the in-plane *a* and *b* lattice vectors were kept fixed to the values determined during a MD simulation at 300K.

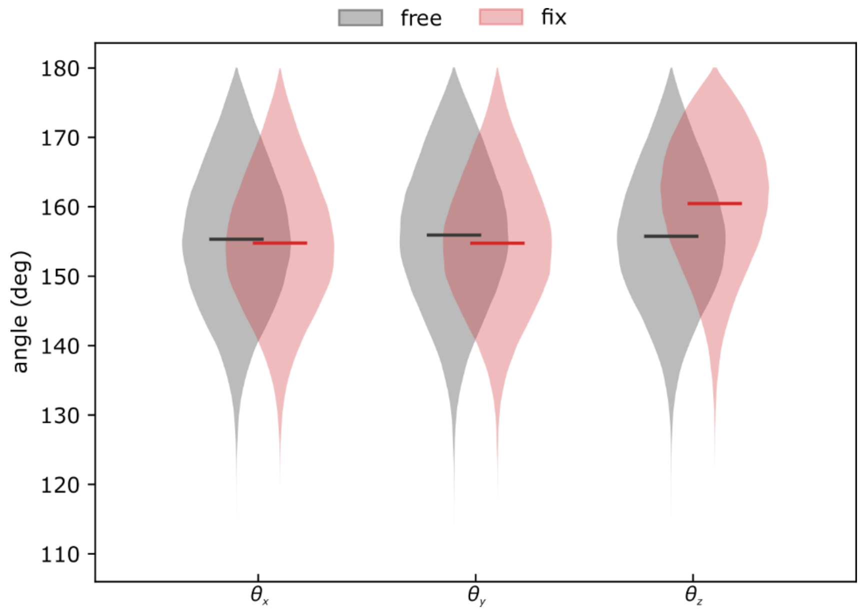

Fig. S6.

Distribution and average value of the Pb-I-Pb bond angles oriented in the *x*, *y*, and *z* directions averaged over all layers during MD simulations of a four-layer $CsPbI_3$ slab. The lattice vectors were kept fixed at their 300 K values and the I^- anions at the bottom were either free to move or were fixed.

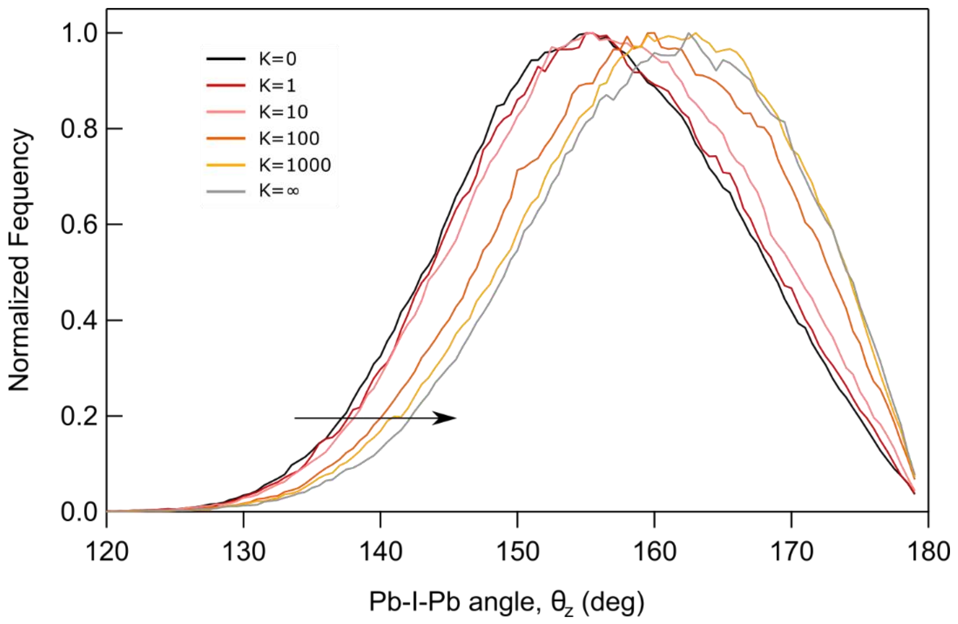

Fig. S7.

Distribution of Pb-I-Pb bond angles within simulated crystals experiencing a varying degree of surface restrain *K*, expressed in kJ mol^{-1} Å^{-2}. The low-angle portion of the distribution becomes heavy skewed with the application of higher restrains (highlight by a horizontal arrow), significantly shifting the tail end of the low-angle shoulder.

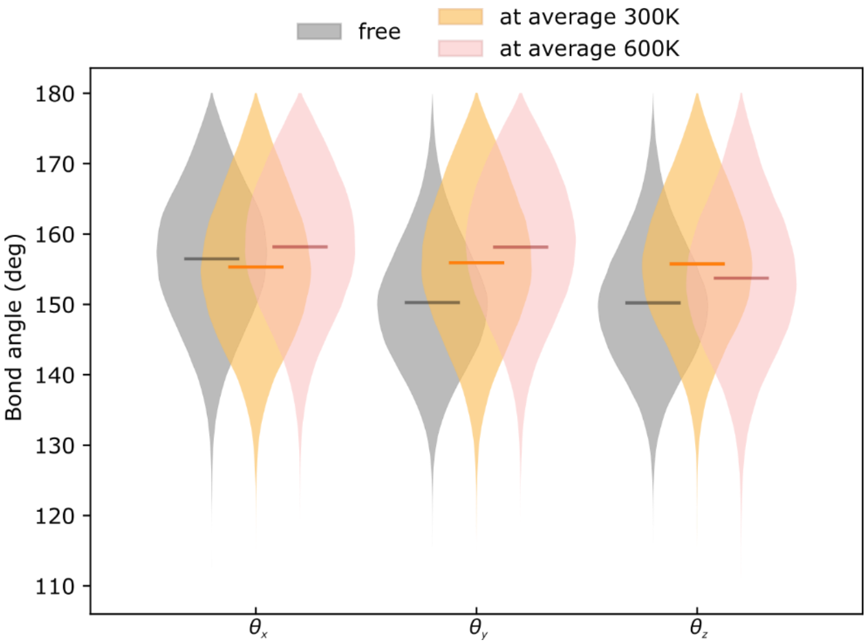

Fig. S8.

Distribution and average value of the Pb-I-Pb bond angles oriented in the *x*, *y*, and *z* directions during MD simulations of a four-layer $CsPbI_3$ slab where the lattice vectors were free or kept fixed at their average value at 300 K or 600 K. The I^- anions could move freely in these simulations. By fixing the lattice vector to values determined at higher temperatures, these calculations mimic the influence of strain due to substrate clamping of a high-temperature processed perovskite thin film which undertakes a much faster thermal expansion rate than the underlying transparent substrate (*1*) (i.e. glass).

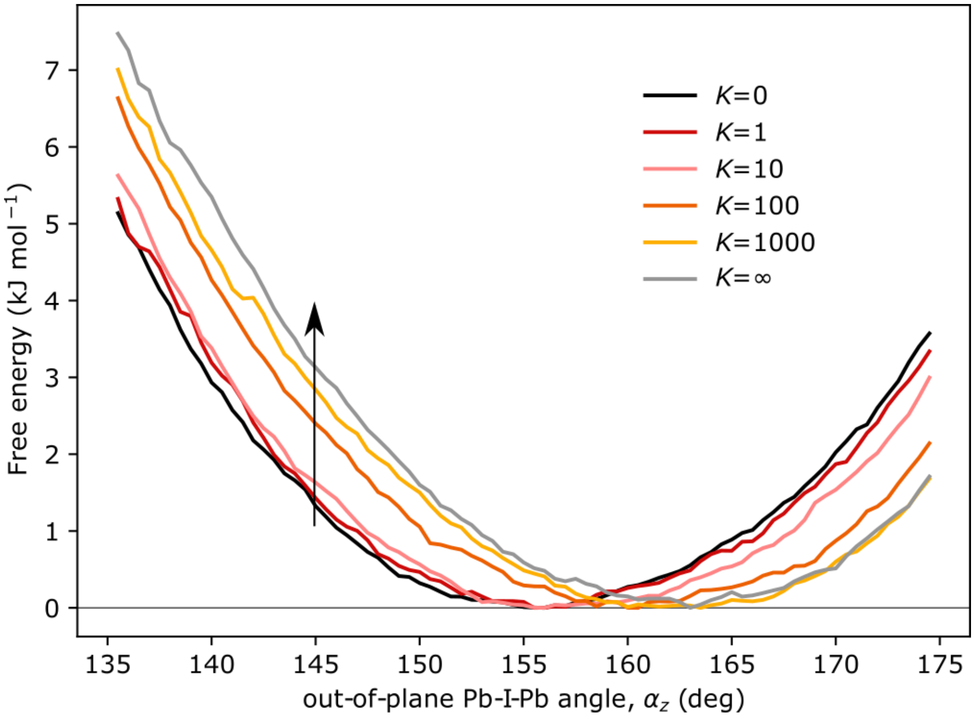

Fig. S9.

Free energy as a function of the average out-of-plane Pb-I-Pb bond angle. The free energy is plotted for different values of K, expressed in kJ mol^{-1} $Å^{-2}$, that define the strength of the restraining potential $V = Kd^2$ of the I^- anions at the bottom of the four-layer $CsPbI_3$ slab. The free energy as a function of the average bond angle is calculated from the probability distribution $P(\theta)$, via $F(\theta) = -k_bT \ \ln P(\theta)$. Here T is temperature and k_b represents the Boltzmann constant. For small angles (α_z<150°), the free energy difference compared to the ground state increases when K increases (highlight by a vertical arrow), indicating that it becomes less probable for the system to transition to the yellow phase structure.

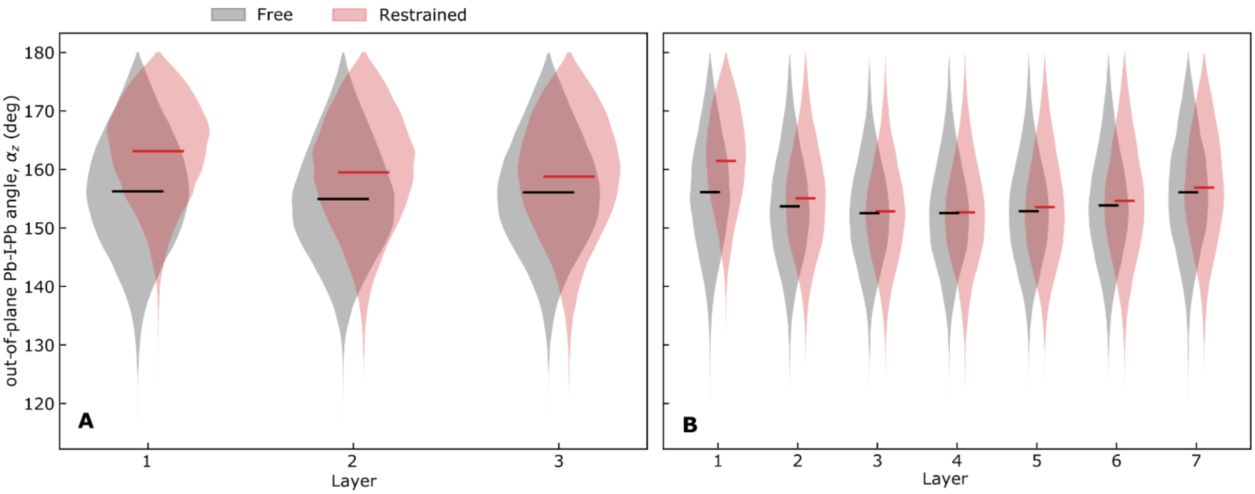

Fig. S10.

Distribution and average value of the out-of-plane Pb-I-Pb bond angles during MD simulations of (A) four-layer and (B) eight-layer $CsPbI_3$ slabs, as a function of the distance with respect to the interface (layer 0). The lattice vectors were kept fixed at their 300 K values and the I^- anions at the bottom were either free to move or were restrained.

Supplementary Note 3. Thermal and photo-thermal decomposition of $CsPbI_3$

In situ GIWAXS studies of high-temperature decomposition in thin film $CsPbI_3$: The thermal decomposition of $CsPbI_3$ was tracked using *in situ* GIWAXS (Fig. S12). The high temperatures were ramp in two different stages using a heated N_2 gas blower, with the slower ramp (5 °C/min) persisting during the material decomposition. Using the different scattering signals which arise during the high temperatures a species evolution t-T plot is derived (Fig. S12B). PbI_2 forms relatively early in the evolution, explaining why use of high temperatures (330 °C) to access the perovskite phase in the thin films inevitably introduces trace amounts of endogenous PbI_2 into the black thin film (Fig. S13).

Photo-thermal decomposition of bulk $CsPbI_3$ powders: Intense, focused 532 nm laser light implemented in a Raman backscattering microscope was used to promote the laser heating in bulk, free-standing $CsPbI_3$ crystals. For this study, we choose laser light which is below the bandgap of δ-$CsPbI_3$ (Fig. S14) to slow down the energy transfer process, and better resolved the laser heating evolution. Further, bulk powders allowed for more intense Raman signals and thus clearer characterization of the expected laser heated products (Fig. S15). Given that 532 nm laser light is used for excitation (photon energy=2.3 eV), the photolytically activated influences (photon-induced bond breaking and chemical decomposition) are assumed negligible compared to the thermally driven process (radiative transfer). After each irradiation dose (scaled using both laser power and time parameters), the subsequent decomposition was recorded via Raman scattering in the same position using low laser power. High intensity exposures notably made the collection of $CsPbI_3$ needle-like material partially ablate and turn black (Fig. S17).

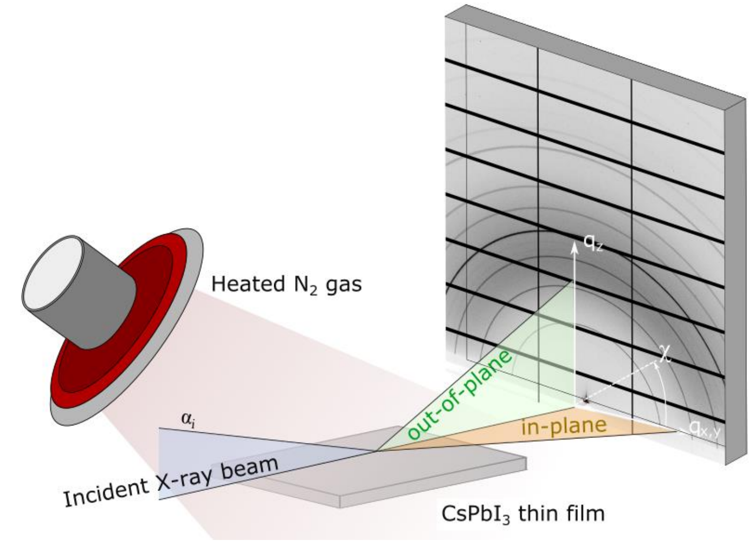

Fig. S11.

Schematic illustration of the scattering geometry of synchrotron-based GIWAXS measurements performed on $CsPbI_3$ thin films. The incident X-ray beam ($\lambda = 0.95774$ Å) scatters from the sample under a grazing angle (α_i), projecting ring-shaped diffraction signals onto the larger-area imaging detector which can sample with a high temporal resolution (0.1 - 0.4 frame/s). The sample temperature is controlled through its immersion at the centre of a heated N_2 gas flow which has been calibrated using a reference silver crystal. Depending on the polycrystalline texture and anisotropic (micro)structure (i.e. biaxial strain), the in-plane ($\boldsymbol{q}_{xy}$) and out-of-plane ($\boldsymbol{q}_z$) scattering vectors and intensities may differ, differences which are resolved by selectively evaluating the relevant scattering axes in the azimuthal domain, χ. Only scattering signals derived from integrating over the whole image (i.e. q_{xyz}) are used for structural refinements.

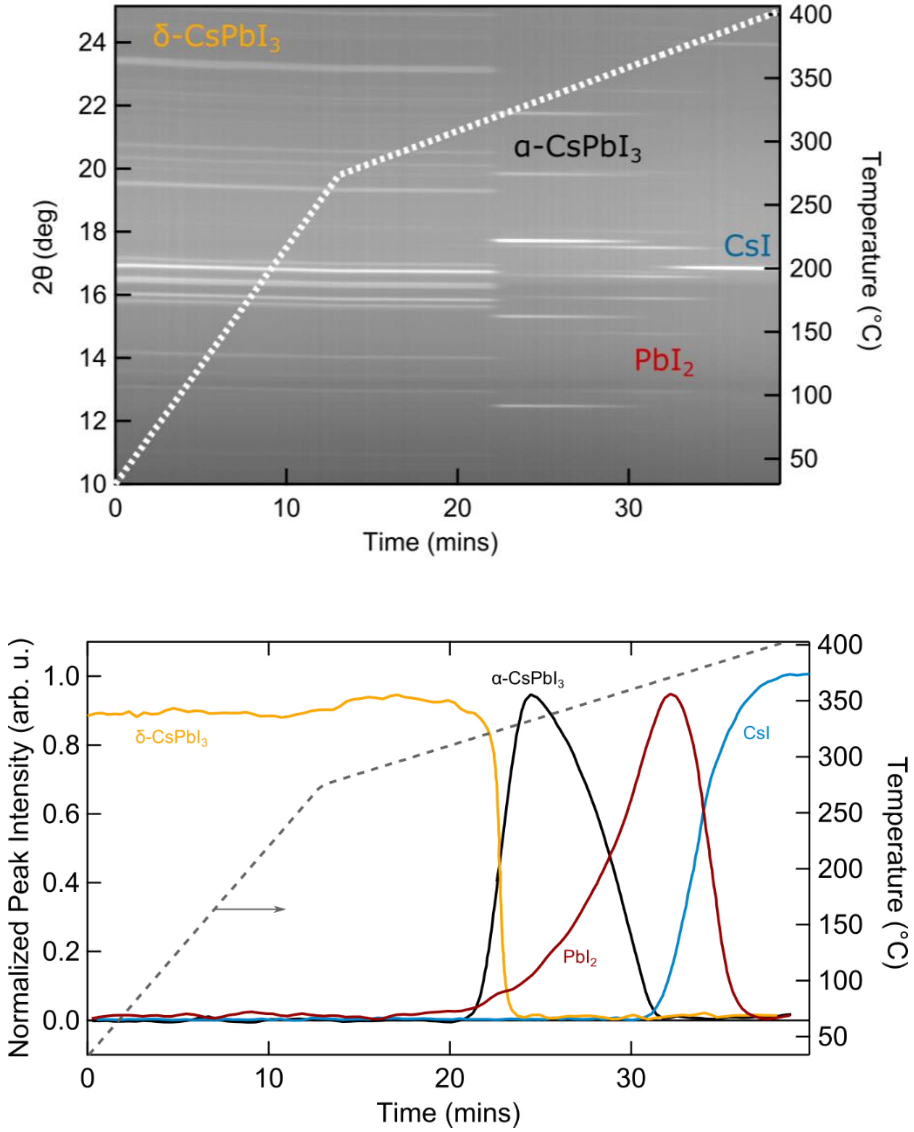

Fig. S12.

GIWAXS t-T profile (***q***$_{x,y,z}$) of $CsPbI_3$ thin film through a high-temperature yellow to black phase transition, followed by a further increase in temperature toward material degradation. The start of the first heating is roughly 30 °C/min and the second is 5 °C/min. Top: waterfall plot of scattering pattern. Bottom: Intensity profile of identified degrading species.

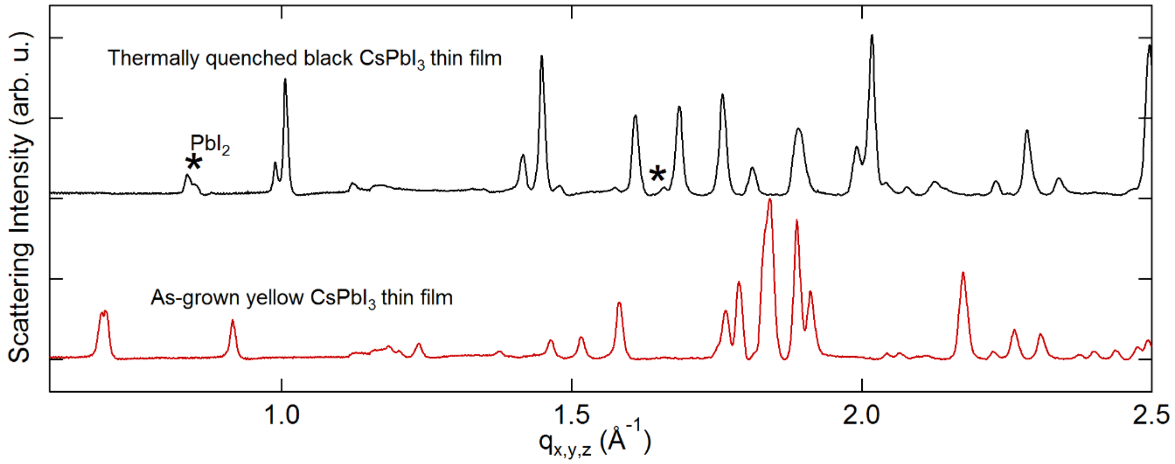

Fig. S13.

Comparison of GIWAXS scans recorded from a $CsPbI_3$ thin films before and after thermal quenching (annealing temperature of 330°C for 1 minute), highlighting (*) the detection of new PbI_2-related peaks. These data have been baseline corrected and normalized, for clarity.

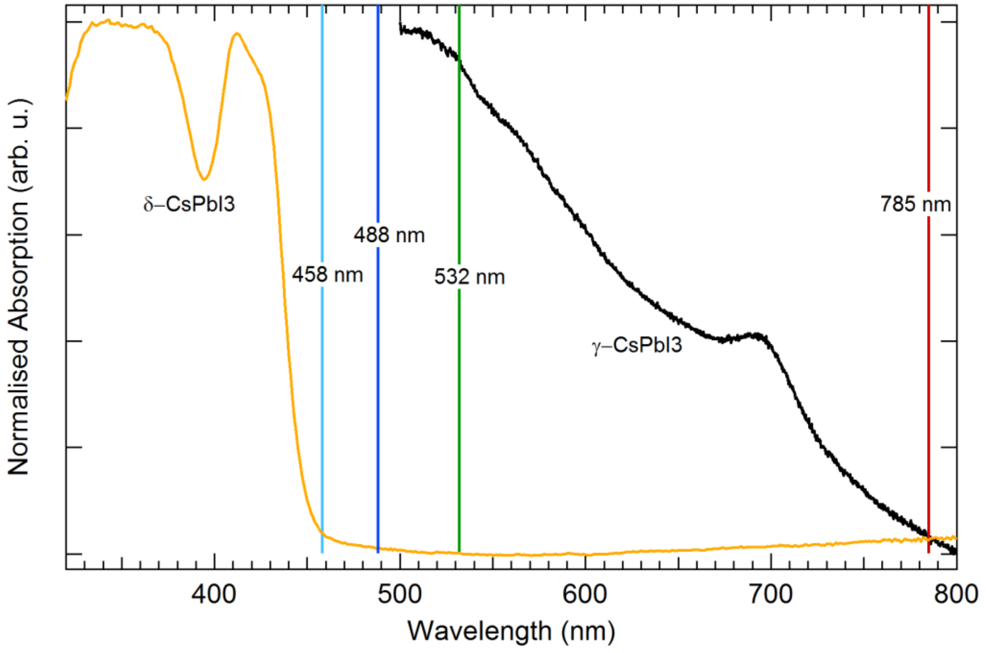

Fig. S14.

Absorption spectra recorded in reflection mode from the surface of yellow and black $CsPbI_3$ thin films, along with the relative position of various excitation wavelengths used in throughout the work for comparison.

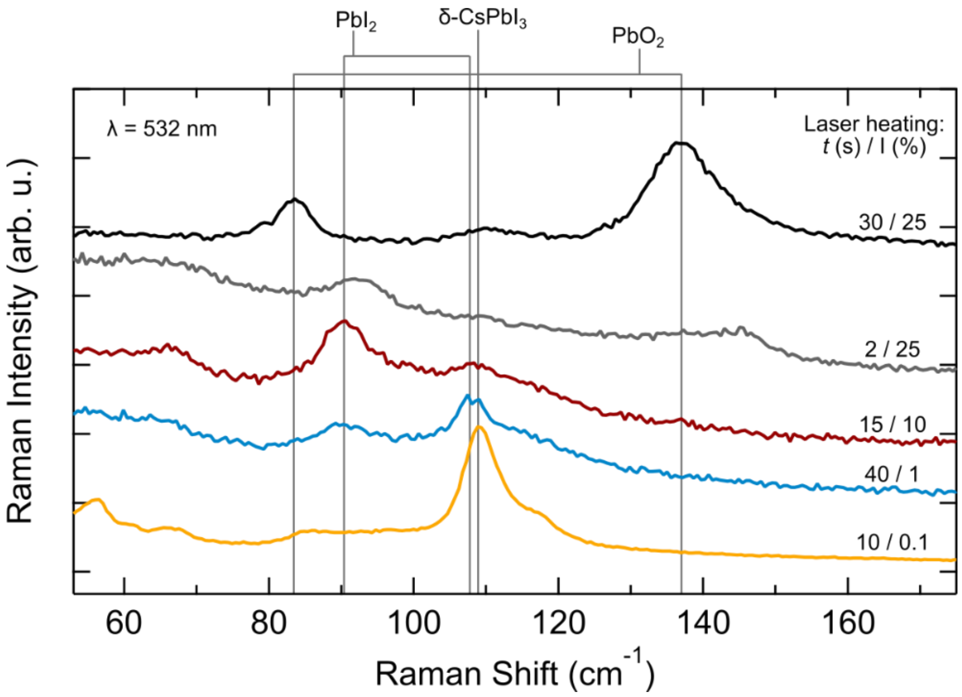

Fig. S15.

Power dependence of laser-induced surface heating of bulk δ-$CsPbI_3$ powders using 532 nm excitation source (10 mW) focused with a 0.25 NA objective, with the laser dose parametrized by time and reduced power faction (controlled by ND filters). Raman spectra are collected using low 532 nm laser power (<0.1%) from the sample surface after exposure to laser heating dose. These data have been normalized and offset, with the vertical lines providing mode assignment (pure PbI_2 and PbO_2; Figure S16). Under an ambient atmosphere, δ-$CsPbI_3$ first decomposes into PbI_2 and then forms a dark ablated lead oxide at higher laser powers.

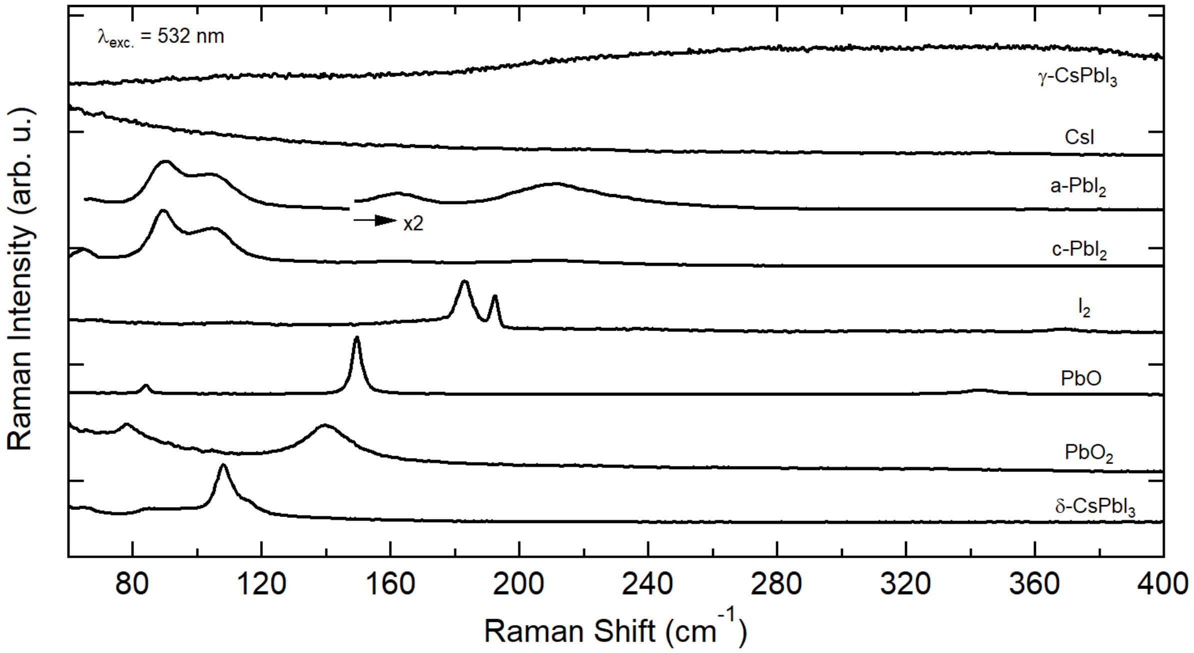

Fig. S16.

Normalized RT Raman scattering spectra of potential degradation products of $CsPbI_3$ when heated in the presence of ambient oxygen. Note the Raman scattering signal from RT black γ-$CsPbI_3$ yields a broad luminescent background using 532 nm excitation, rather than clear Raman active bands. Likewise, the ionic CsI crystal only exhibit a strong central quasi-elastic band and is also included for completeness.

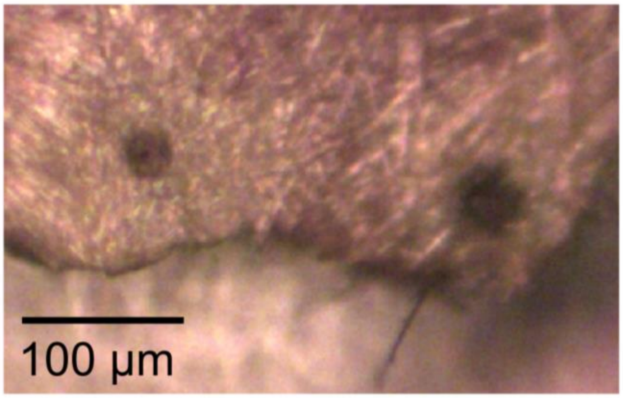

Fig. S17.

Optical image recorded after bulk $CsPbI_3$ powder is exposed to high power 532 nm laser irradiation two times (dark circular regions to the left and right).

Supplementary Note 4. Photolithographic patterning of thin films and examination of the physical changes

Controlled photolithographic patterning: As-grown $CsPbI_3$ thin film surfaces were laser processed using above bandgap 458 nm cw-laser light for efficient energy transfer and the ability to rapidly pattern (10 mm/s) and minimize processing footprint across the final optoelectronic thin film surface. A laser microscope was used for the patterning (simplified scheme shown in Fig. S18). To confirm that the Gaussian microprobe spot sizes neared the diffraction limit, an optical beam expander was placed before the microscope optics used to focus light onto the sample (UPLXAPO10X ×10, 0.4NA objective). The motorized XYZ stage (Märzhäuser Wetzlar) of the microscope enabled precise sample manipulation and laser light was visualized to focus on the sample surface through a video camera/beam splitter assembly. With the thin film surfaced levelled relative the processing plane, the laser spot was moved and translated by a series of XYZ coordinates controlled by a computer, with a patterning speed of 10 mm/s. The power density of the focused laser spot was controlled via a laser current supply module and selecting the appropriate neutral-density filter on a computer-controlled wheel and was measured using a calibrated power meter (ThorLabs photodiode S130VC). A Gaussian shaped incident beam is confirmed via scattering across the edge of cleaved Si wafer and the power density values were calculated for the $1/e^2$ (13.5% of peak) beam diameter.

Photolithography power dependence: Microgrids are fabricated using different laser power and their topologies are recorded using AFM (Fig. S19). The degree of structural ablation on the thin film surface is evaluated by line scans taken across micro-processed tracks. A photolithographic pattern can be formed without significantly oxidizing/ablating the film surface using a laser power density of 300 W/cm^2 (at 10 mm/s), which is implemented to micropattern thin films in the main text.

Local phase kinetics of square array: A sequence of 50 μm squares were patterned on the thin film surface using a laser power density of 300 W/cm^2 (10 mm/s). The films were then thermally quenched and monitored in an ambient environment using an optical microscope.

Raman spectroscopy characterization: Raman microscopy (785 nm excitation) is used to probe three areas across the microprocessed area in Fig. 2B of the main text. The results are compared to relevant reference data; in situ high-temperature Raman spectroscopy (Fig. S21), DFT calculations (Fig. S22), and the Raman spectra of pure products (Fig. S23). An expansion of the RT black-phase Raman spectrum recorded from the stable interior of the microprocessed area (Fig. S24) yields fourth-order Raman modes. These high-energy vibrational signatures are also used for phase identification but are not captured in the first-order Raman DFT calculation (Figure S22).

GIWAXS characterization of δ-$CsPbI_3$ with embedded microgrid: A section of a δ-$CsPbI_3$ thin film was laser patterned (5×5 mm^2) area using a relatively tight square grid spacing of 10 μm to maximize microgrid-related structural signals. GIWAXS recorded on and off the microgrid

verifies PbI_2-related signals to contribute to the scattering pattern recorded from micro-processed δ-$CsPbI_3$ thin films (Figure S25).

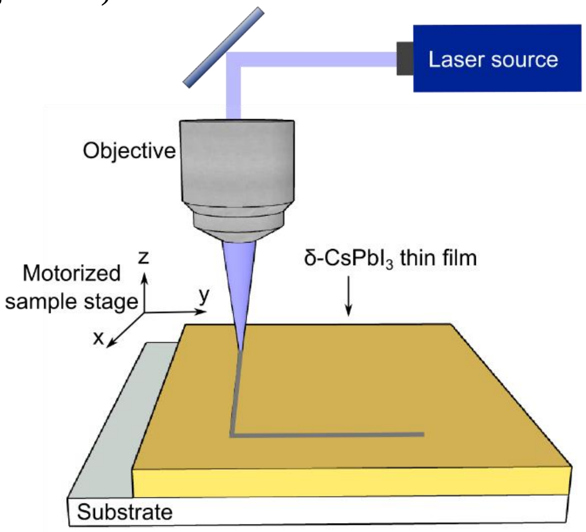

Fig. S18.

Schematic diagram of the laser processing instrument used. The sample area is exposed to an ambient environment. An objective with relatively low magnification and NA (0.4) is used to focus 458 nm laser light in the absence of z-tracking. This increases the depth of field and overall tolerance to changes in height across large film areas.

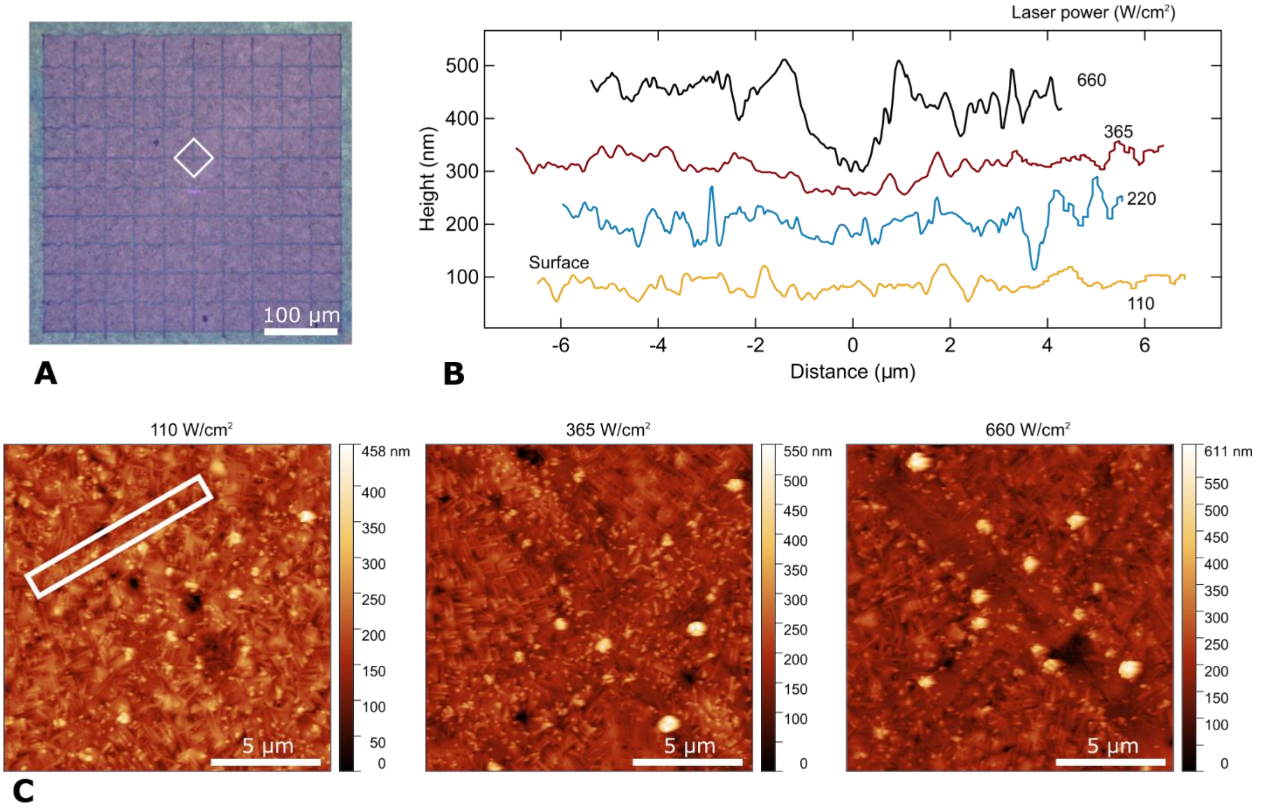

Fig. S19.

(A) Optical image of microgrid area after annealing patterned using a 458 nm laser power density of 300 W/cm^2 (10 mm/s). The white diamond shape shows a typical AFM map area, imaging the intersection of orthogonal lines in the grid. (B) Examples of AFM line scans recorded across photolithography tracks made using different laser power densities. (C) Example images of intersecting microgrid lines, with the white rectangle showing a typical line scan area across the microgrid track, forming the data presented in (B).

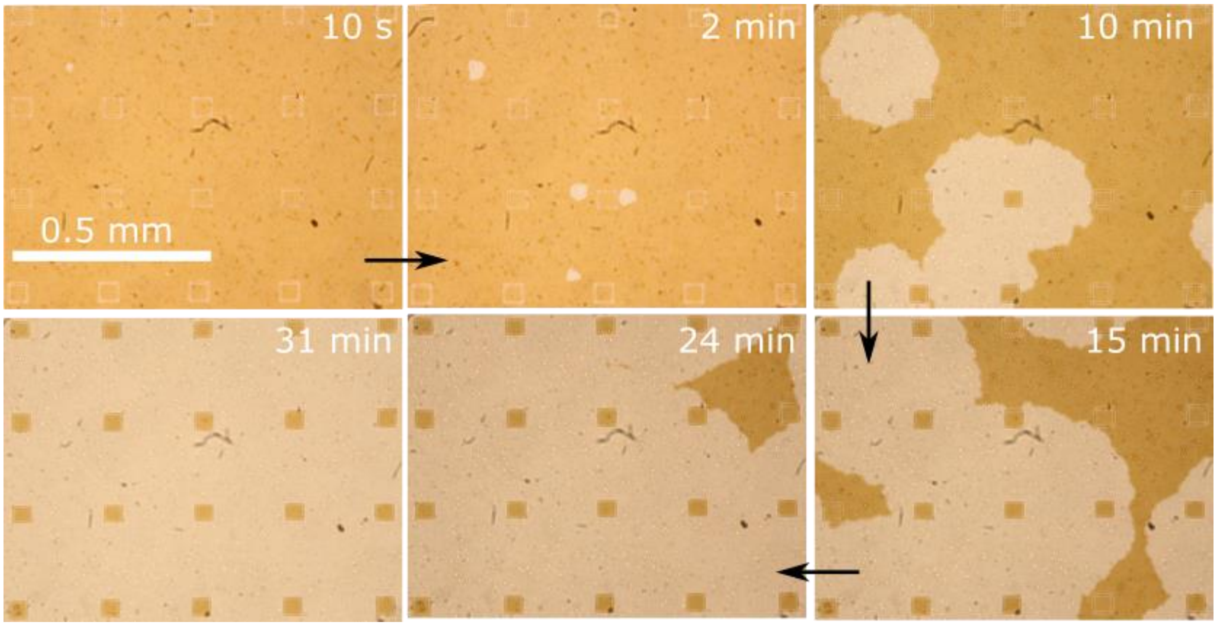

Fig. S20.

Optical images recorded under an ambient atmosphere (42% RH) after thermally quenching a 50 μm square micro-processed array, which is the wider view of the data shown in Figure 2D.

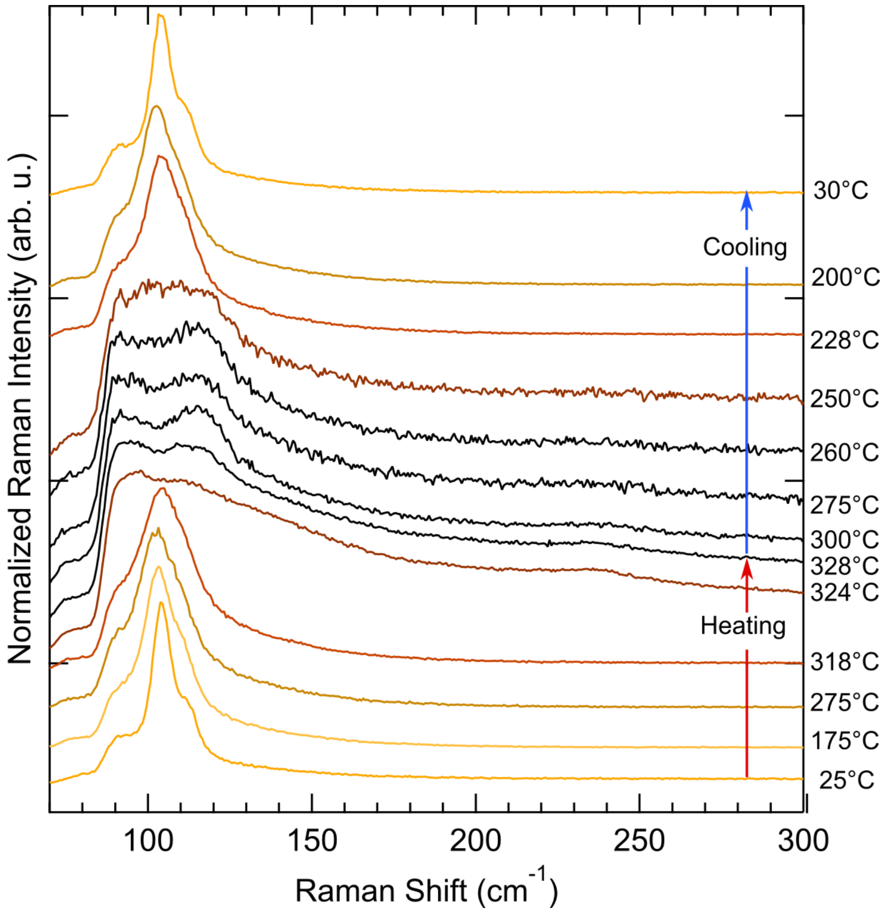

Fig. S21.

Raman spectra (785 nm) recorded in situ from bulk $CsPbI_3$ as it is heated to its black phase and then cooled back to RT, returning it to its thermodynamically preferred yellow phase.

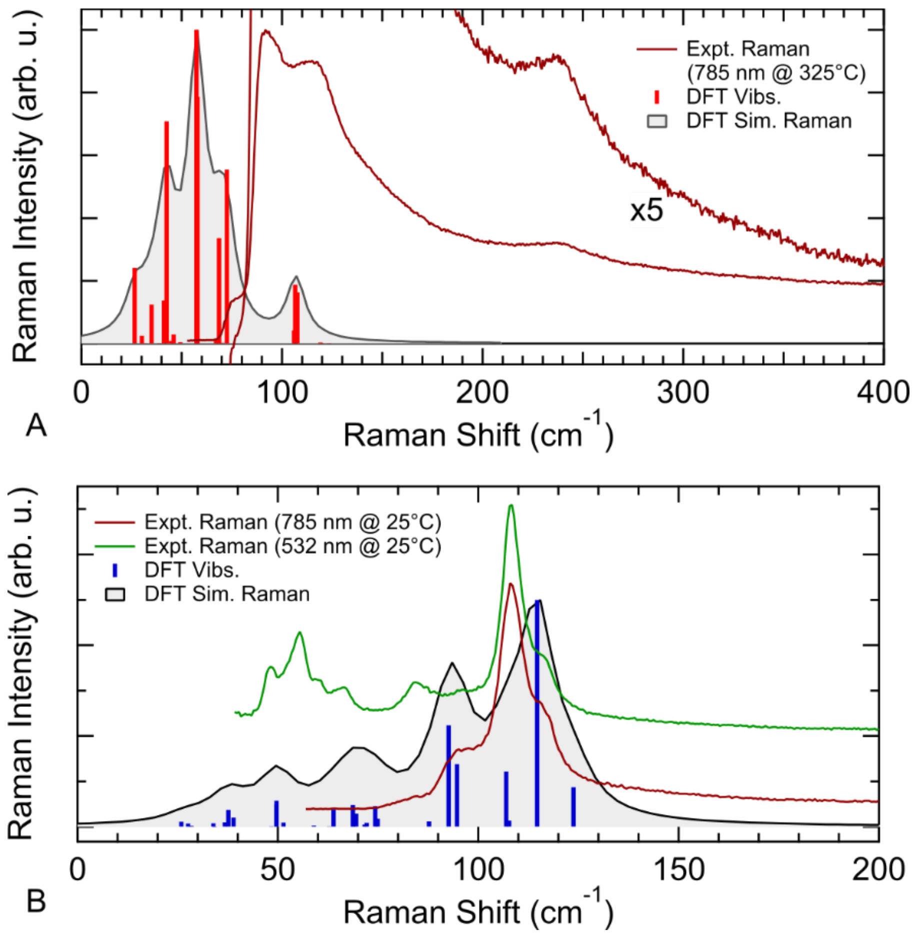

Fig. S22.

Comparison of normalized experimental and calculated Raman spectra. (A) Raman scattering spectrum of the high-temperature bulk $CsPbI_3$ powders while it is in the black phase at 325°C, using 785 nm excitation. The rescaled trace highlights the high-energy Raman mode seen near 250 cm-1, which far above the first order spectrum predicted by DFT. (B) Raman scattering spectrum of the RT bulk $CsPbI_3$ powders while it is in the yellow phase, using both 532 nm and 785 nm excitation. The simulated Raman modes and spectra are generated using a broadening function (fwhm = 10 cm^{-1}), and rescaled using a factor of 1.1 to align their frequencies with the experimentally observed spectra.

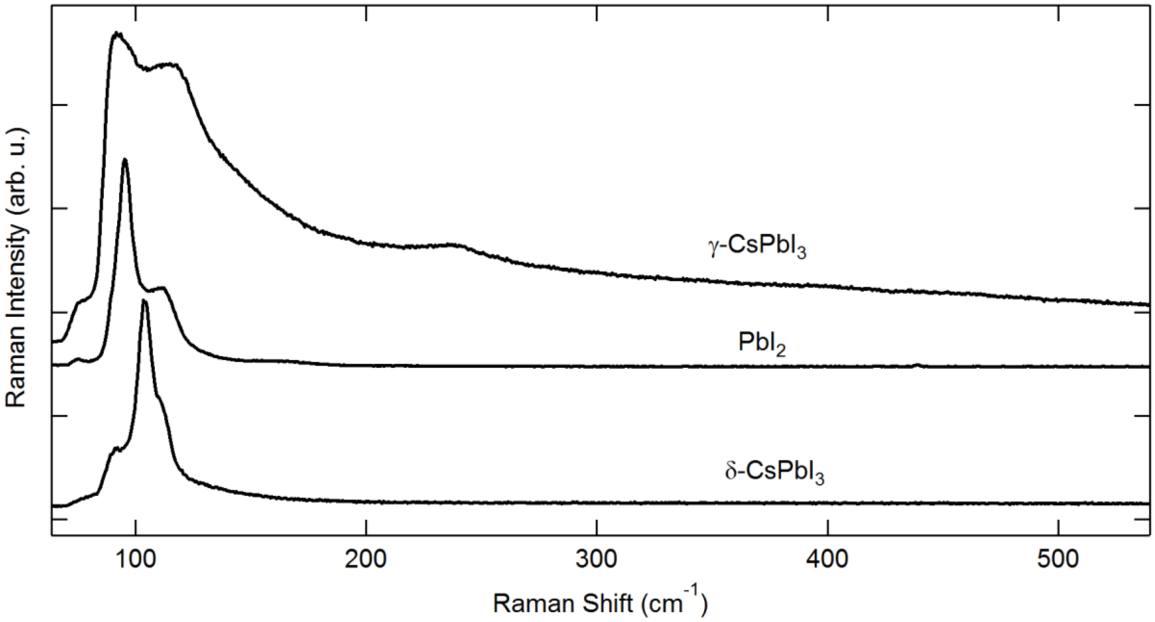

Fig. S23.

Normalized Raman scattering spectra of yellow and black phase $CsPbI_3$, and PbI_2, using 785 nm excitation.

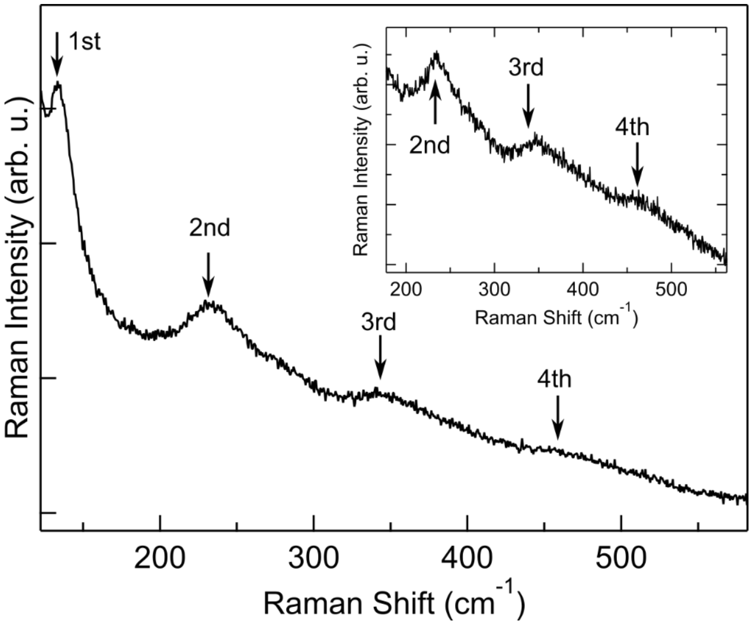

Fig. S24.

A rescaling of the Raman spectrum recorded from the stabilised black phase depicted in Figure 2b of the main text. The observation of prominent higher order Raman scattering of optical modes is highlighted in the inset, up to a fourth-order mode. The second-order mode here is used to further verify the presence of black perovskite phase within the stabilized microgrid.

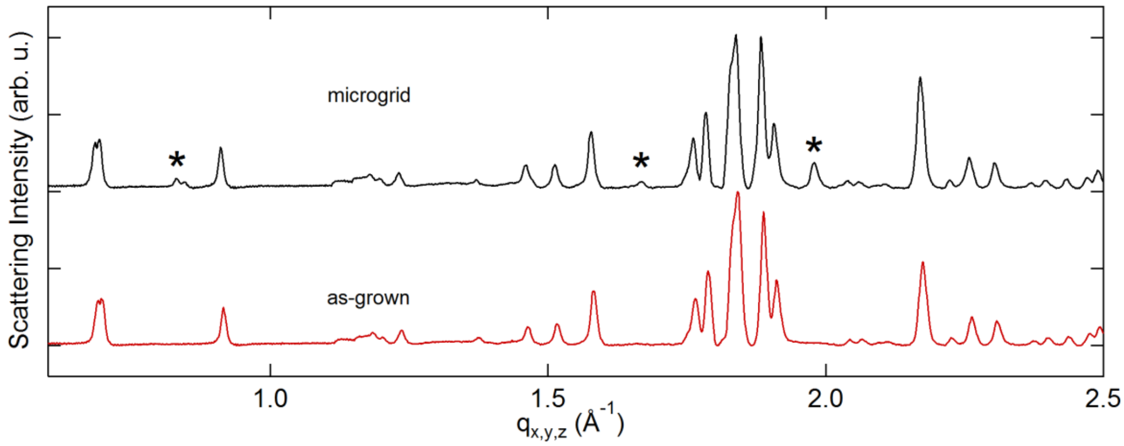

Fig. S25.

Comparison of GIWAXS scans recorded from a yellow $CsPbI_3$ thin films on and off a laser processed area (a large-area microgrid with a spacing of 10 µm). Peaks are highlighted (*) to show the detection of PbI_2-related signals. These data have been baseline corrected and normalized, for clarity.

Supplementary Note 5. Size-dependent phase kinetics of microgrid sample stability

Size-dependence of microgrid phase stability: Photolithographic patterns of 14×14 gridded squares make up small-scale areas for testing the size-dependent stability of the microgrid. In this way, each sampled area has a 2 square buffer left on the perimeter which is not considered and the interior 10×10 squares are evaluated and counted out of 100 over time. For each grid size, the stability of corresponding patterns is studied while stored over time under an unregulated ambient atmosphere (between 42-48%RH for these experiments). With decreasing size, the microgrid becomes more stable, as shown in the size-time image matrix in Fig. S26.

The fraction of decaying grid squares with time are used to develop Avrami plots for the black to yellow phase transformation. This is compared to as-grown thin films (Fig. S27). As outlined in the main text, two regimes govern transformations of the laser patterned thin films; early on the processed films follow the trend of the as-grown samples (regime I) and later on the microgrid quenches further spread of the phase decay (regime II). The overall system is limited to its lowest activation barrier, given that spatial heterogeneity exists across the grainy thin film surface and a yellow phase wave front can easily propagate inter-granularly once triggered. With an approximately constant nucleation density (per unit area) across the thin film a large portion of the supressed reaction is related to the decreasing volume in which each nucleation event can spread and transform the surrounding film, due to the structural isolation of each square.

In situ fluorescent microscopy of microgrid phase stability: To better visualize the phase evolution across a decaying black-phase thin film, optical transmission and fluorescence images (488 nm) were recorded *in situ* while a processed film is slowly heated to 330°C and then cooled under an ambient atmosphere (Fig. S28). The film is tracked for over roughly an hour (under constant scanning 488 nm laser) showing non-local phase behavior near the microgrid shortly after cooling. The processed tracks distinctly interrupt the normal evolution of the phase decay, helping to stabilize regions of the film sometimes up to millimeters away from gridded areas, while remaining linked by continuous regions of black perovskite phase.

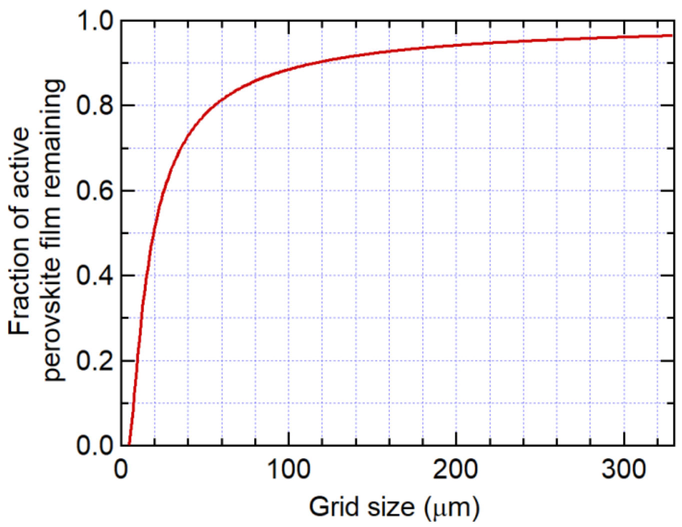

Fig. S26.

Calculated fraction of optically active perovskite phase remaining after micropatterning with a footprint of 4 μm, as a function of the microgrid size.

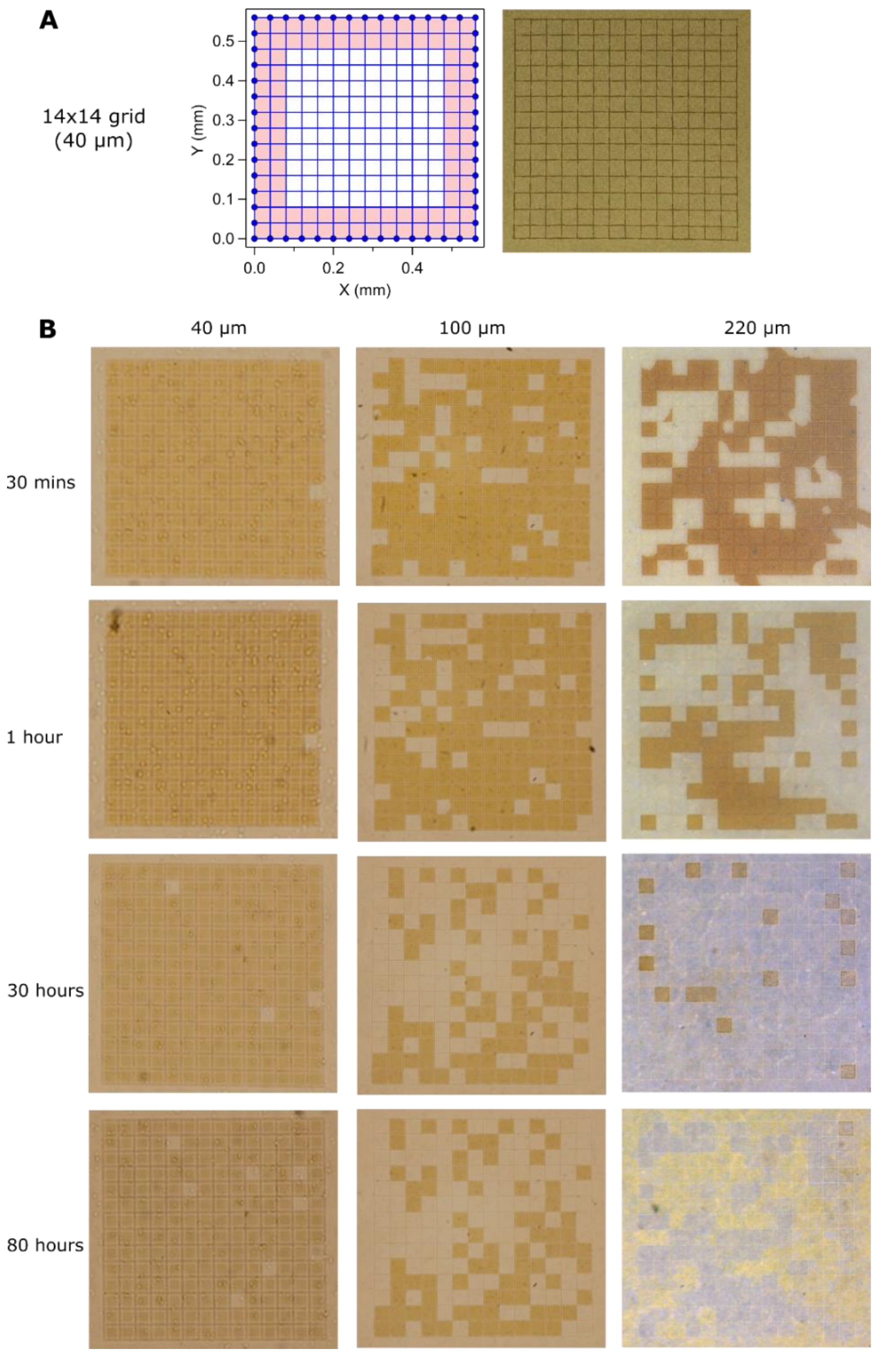

Fig. S27.

(A) Example of test grid. Left: Coordinates of a micro-processed 14×14 grid made of 40 µm squares, with the red shaded area representing the 2 square buffer between the interior used for statistical counting of grid survival (inner 10×10 portion). Right: Optical image of an example of an as-grown $CsPbI_3$ thin film which has been processed with a 14×14 grid using 40 µm squares. (B) Image matrix of quenched $CsPbI_3$ thin film areas processed with varying grid sizes and stored over time under ambient conditions (42-48%RH).

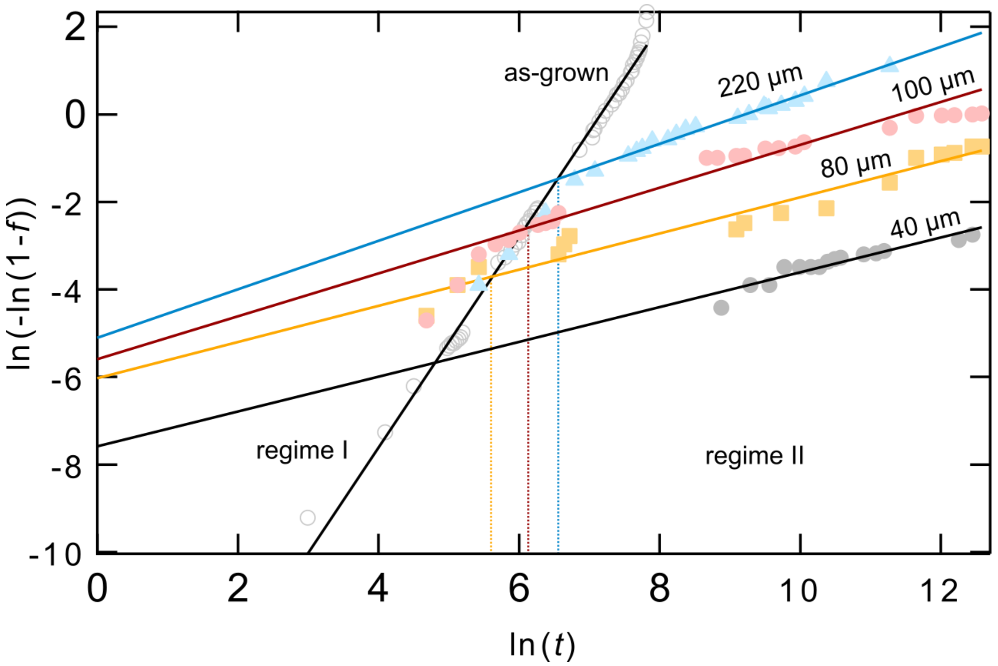

Fig. S28.

Avrami plots for the black to yellow phase transformation in as-grown and micro-processed thin films using different grid dimensions. The linear fits to these plots are made for ln(*t*) values higher than the as-grown trend line (in regime II), given that before this time (i.e. before the broken vertical lines) the boundary of the grid on average has not yet inhibited the spread of the yellow phase.

In regime II, the changing fraction of transformed yellow phase (*f*) exhibits Avrami-like behavior, given that linear trends are exhibited with time (i.e. unchanging dynamics). The linear fit here is governed by the Johnson-Mehl-Avrami-Kolmogorov (JMAK) equation: $\ln[-\ln(1-f)] = \ln(k) + n\times\ln(t)$. Thus, the y-intercept, $\ln(t) = 0$, of the Avrami plot yields the logarithm of the rate constant, *k*, which are compared in Fig. 3d of the main text.

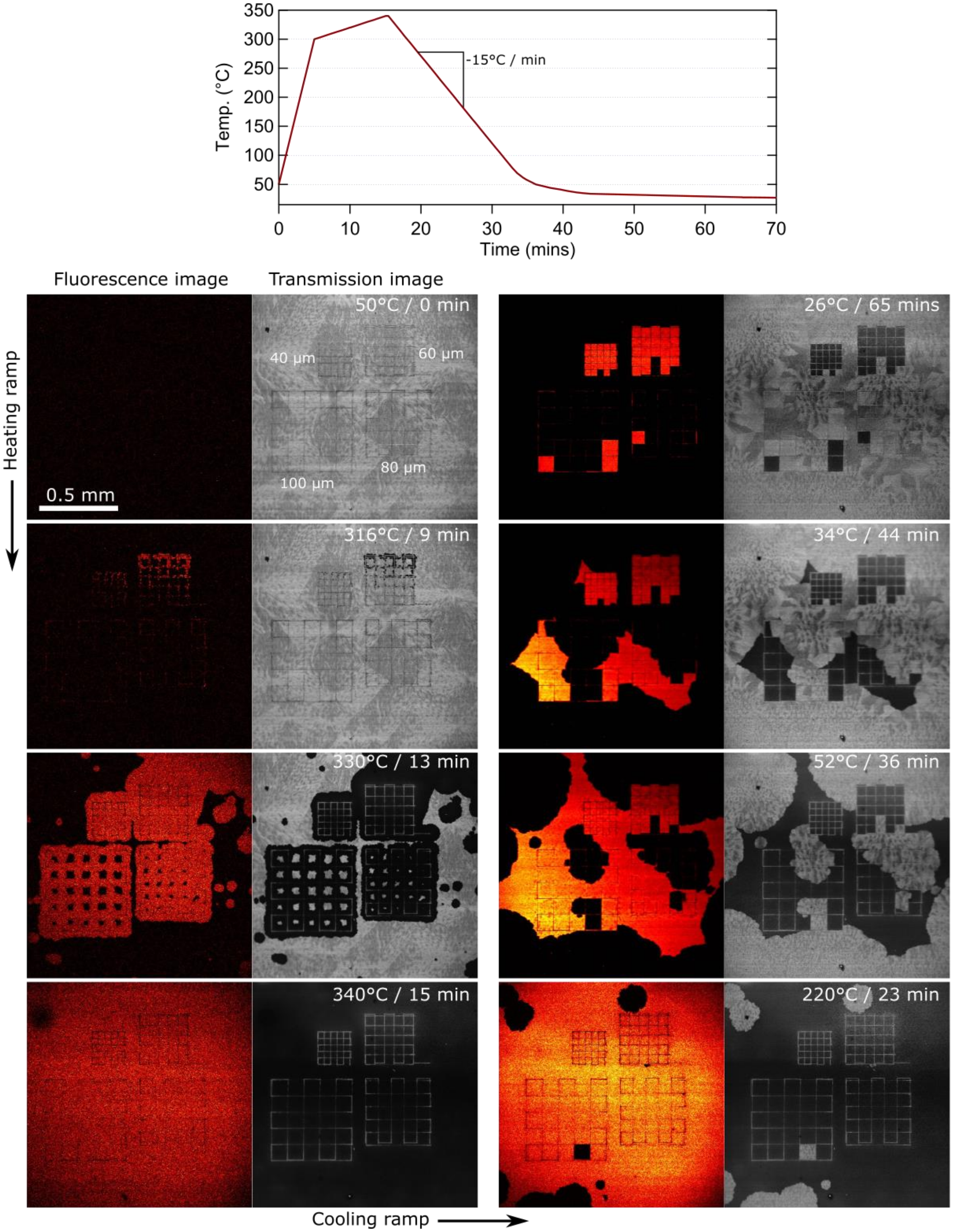

Fig. S29.

Corresponding fluorescence and transmission (λ = 488 nm) images (bottom) of a micro-processed $CsPbI_3$ thin film recorded under an ambient atmosphere (~52% RH) at different temperatures during a high temperature annealing profile (top). The different square sizes (each forming 5×5 grids) is indicated in the first transmission image.

Supplementary Note 6. Long-term stability of microprocessed thin films

Long-term stability test: To maximize the remaining area of the optically active film surface, long-term stability testing is performed on 40 µm grids, which leaves ~73% of the film optically active when patterned using a processing footprint of ~4 µm (i.e. the width of the photolithography track). Processed thin films exhibit a strong resistance to ambient conditions compared to their as-grown counterparts. To quantify this over time, the stability of 10×10 grids were evaluated after thermal quenching the black phase. Samples were stored in both ambient (Fig. S29) and dry atmospheres (Fig. 30; samples were sealed in an dry optical imaging mount and held in larger desiccator) and periodically imaged in an optical microscope. Light shielding was not implemented and the samples were left on benchtops of the laboratory (in Leuven, Belgium). For ambient storage, the unregulated RH varied between 25-60% and near day 110 of testing, widespread discoloration (moisture damage; Fig. S31) prevented the study from continuing.

Patterning on device-ready substrates: To confirm that our microgrid stabilizations strategy is a general one, we embedded 40 µm grids into thin film deposited onto ITO substrates (Fig. S32). In this scenario, after 24 hours of ambient storage, the microgrid has prevented phase decay.

Selective destabilization in a black $CsPbI_3$ perovskite microgrid: An important feature of the microgrid is the restricted connectivity of the gridded polycrystalline network, which isolates destabilization events and prevents them spreading. To clearly show this aspect, focused light is used to promote a controlled nucleation event in a stable black microgrid film, akin to a spontaneous nucleation. Optical images then track the subsequent yellow phase evolution as it cascades out from the triggering point, forming a radial-like wave (Fig. S33). The same localized nucleation procedure can be used to control the phase of targeted perovskites blocks across a microgridded film and generate pixelated images (Fig. 3A of main text).

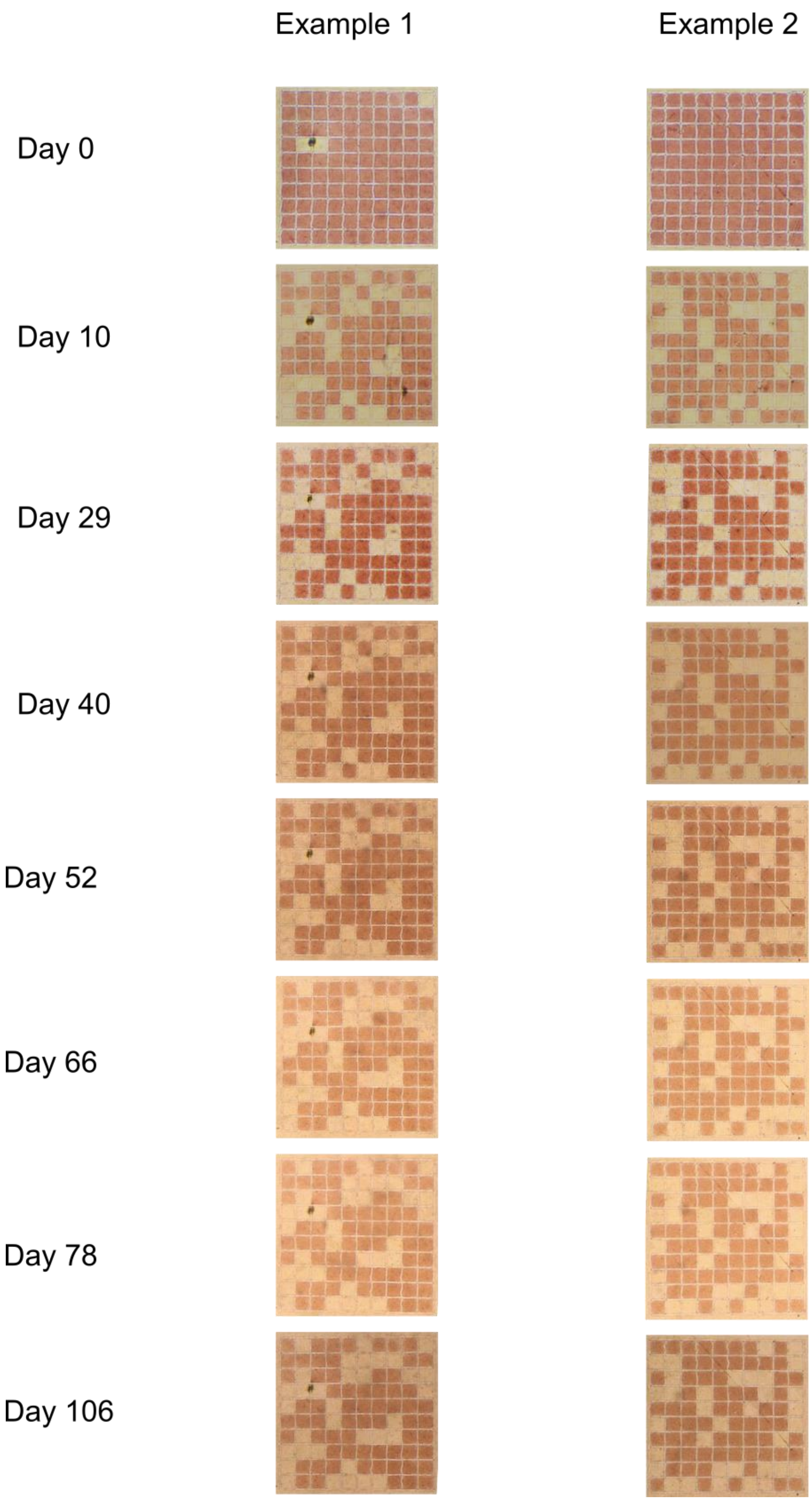

Fig. S30.

Representative images of the 10×10 40 µm squares used to record the phase decay over time under an ambient environment.

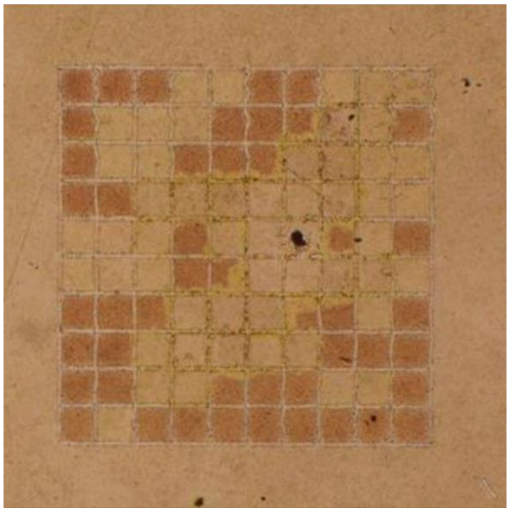

Fig. S31.

Representative optical photograph of a 10×10 grid atop $CsPbI_3$ thin films processed with a 40 µm pattern size, imaged at 110 days after storing in an ambient environment. The middle grid shows moisture damage (i.e. clear discoloration of the initial yellow and black phases), preventing it from being further examined.

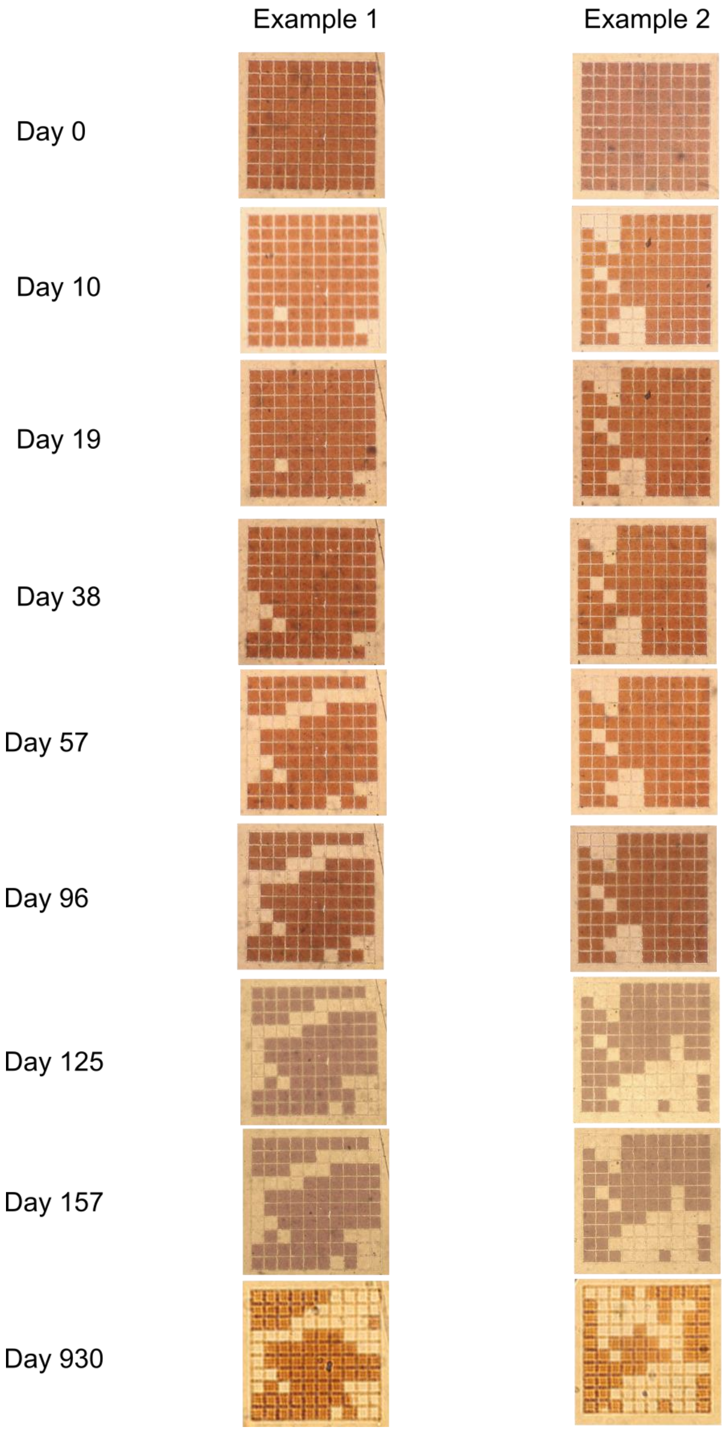

Fig. S32.
Representative images of the 10×10 40 µm squares used to record the phase decay over time under a dry environment.

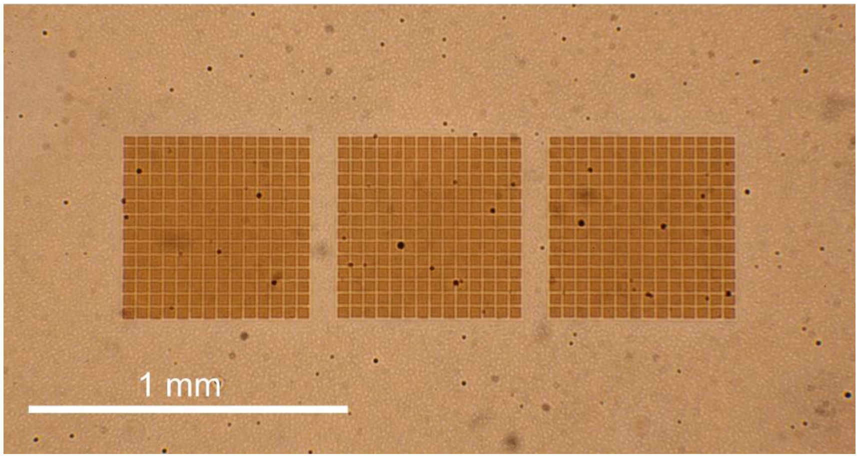

Fig. S33.

Optical images of $CsPbI_3$ thin film spin coat onto ITO covered substrates and processed with three 40 µm grid patterns. The sample was annealed, and the black phase thermally quenched to RT, then stored under an ambient environment (~45% RH) for 24 hours before recording the image.

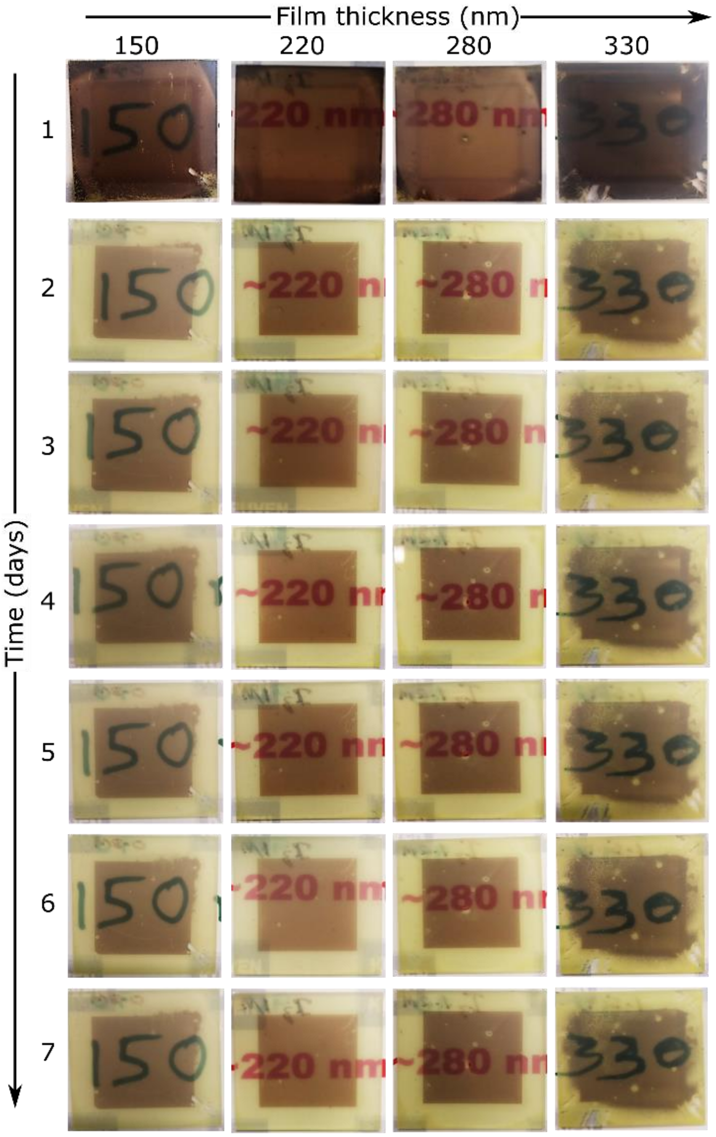

Fig. S34.

Optical images of $CsPbI_3$ thin films growth to different thicknesses. All of the films have an embedded 40 μm grid pattern which stabilizes the interior 4 cm^2 of the film. The films were annealed and then stored under an ambient environment (39-41% RH) for 7 days.

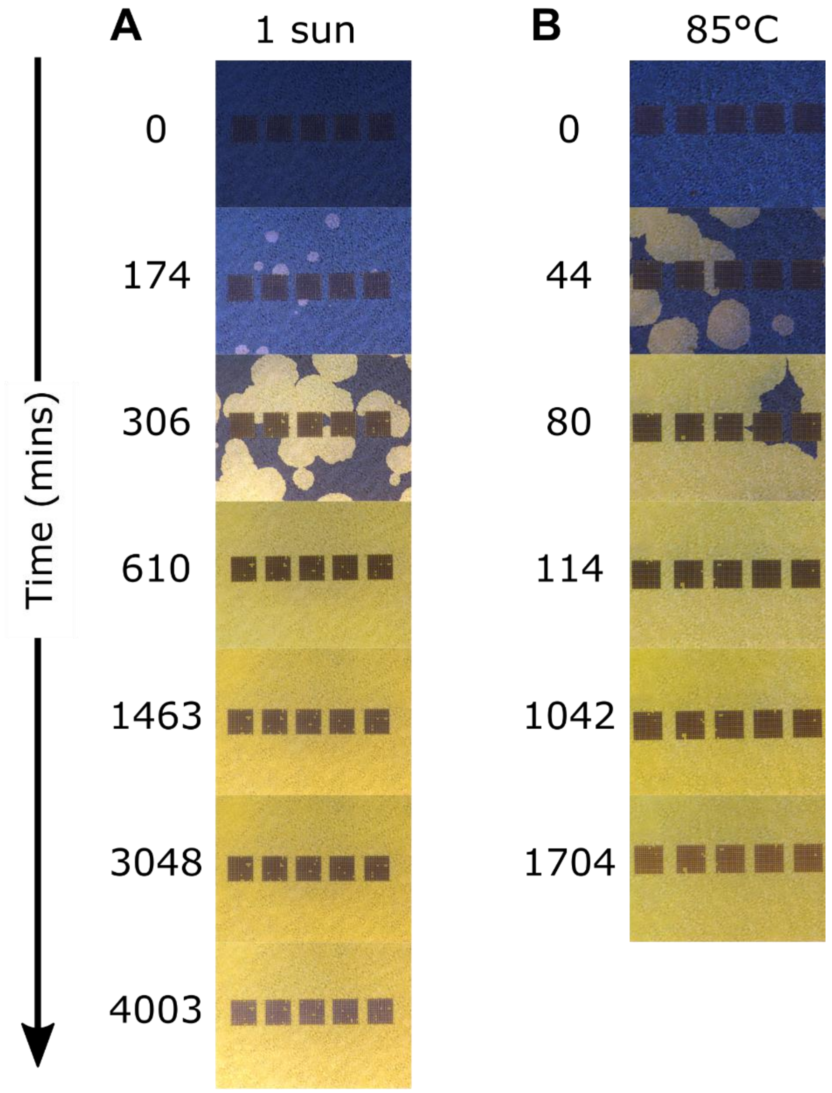

Fig. S35.

Optical microscopy images tracking the stability $CsPbI_3$ thin films fitted with an array of five 14×14 microgrid with a length each of 40 µm. After annealing the films at 335°C for 2.5 minutes, they were then examined under (A) 1 sun, and (B) 85°C. The observations ended when decay of black phase ceased and the system stabilized.

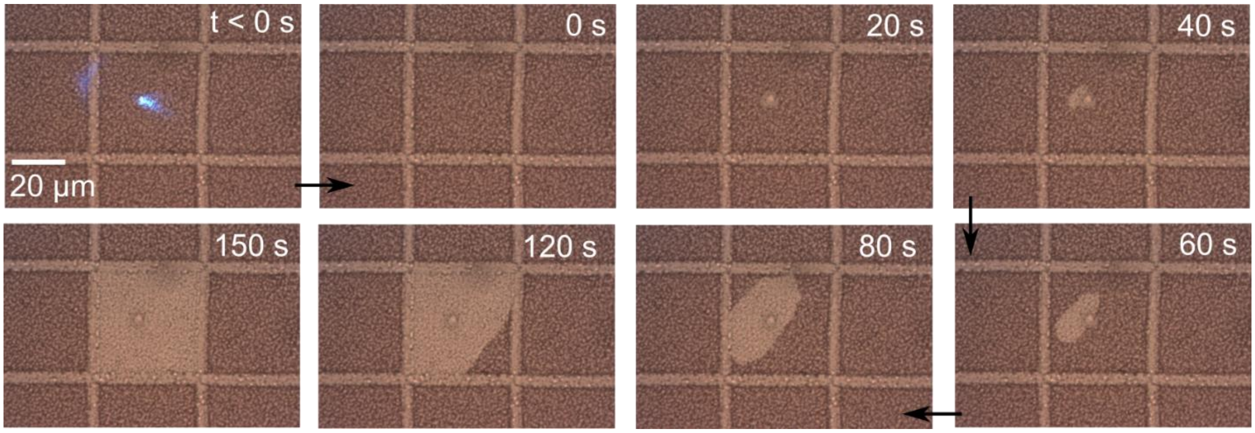

Fig. S36.

Corresponding optical images of the temporal phase evolution ($t \geq 0$ s) after yellow-phase nucleation is triggered by focused laser light (488 nm at ~2kW/cm^2).

Supplementary Note 7. Scaled, large-area patterning and characterization of the embedded interface

Photolithographic patterning at the wafer-scale: The same processing parameters are used to pattern a 2×2 cm^2 microgrid area of a wafer; 40 µm grid size, 458 nm laser excitation with 300 kW/cm2 power and a scanning speed of 10 mm/s. This process took roughly 45 minute per film. A photograph of such a film is shown in Fig. 3B of the main text.

Optical properties of microgrid: High-resolution optical characterization of the perovskite thin film embedded with a microgrid was carried out to see the impact of the introduced interface. Steady-state and time resolved photoluminescence microscopy studies were used to detect changes near the bordering microgrid (Fig. S35). Importantly, no detectable changes were recorded in the stabilized film due to the introduction of the photolithography pattern. This is consistent with the well-known passivating role that additive PbI_2 has on traps in lead iodide perovskites.

Studies of the embedded interface using GIWAXS: Mesostructure across large grain populations, i.e. with respect to their size, shape, and orientation, can arise over lengths of hundreds of nanometers. For synchrotron XRD experiments employing large-area 2D detectors, this information is encoded in the shape, width, and azimuthal angular distribution of Debye-Scherrer diffraction rings. A grazing incidence geometry (Fig. 11) is used to record the scattering signals of black $CsPbI_3$ thin films with and without a microgrid (Fig. S36).

Structural refinement (Le Bail method) confirms their common orthorhombic perovskite phase (*Pbnm*; a = 8.639 Å, b = 8.953 Å, and c = 12.580 Å). However, there are clear differences in the 2D GIWAXS images because of the modified polycrystalline thin film texture, i.e. the relative orientation and distribution of crystal grains. The overall texture expression in the microgrid sample is driven by two anisotropic components. The first arises during annealing where the black perovskite forms a planar interface with the underlying substrate and introduces strain once cooled to RT. As-grown black $CsPbI_3$ thin films thus exhibit well-defined variations their Bragg peak intensities in the azimuthal domain (Fig. S37 and S38). The second texture component is made of intense hot spots in the scattering signal, due to the introduction of the embedded 3D interface. This manifests as narrow, intense rises the Bragg signals presented in the azimuthal domain (Fig. S37 and S38). More examples of the signal hot spots generated by the microgrid are shown in Fig. S39, showing there random character.

Texture analysis: Given the relatively small size of the incident X-ray probe (beam dimensions of 80 × 30 $µm^2$ [H × V]), the anisotropy of the 40 µm squares does not average out to reform a signal akin the as-grown film. Within this experimental regime (i.e. relative size of the incident beam to the microgrid structure), quantitative estimates can be made regarding the population of grains being driven to form a microgrid-induced texture; full details are found in Fig. S40.

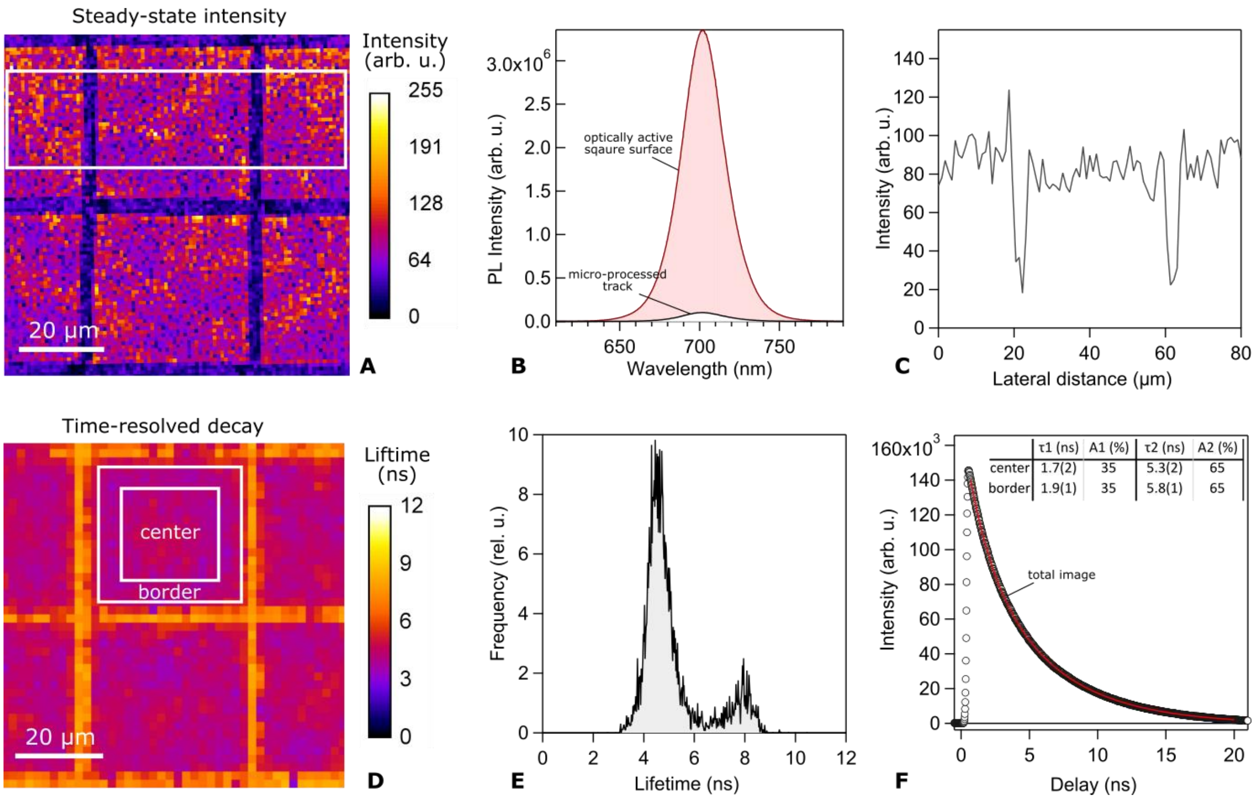

Fig. S37.

(A) Confocal micro-PL intensity map (detection wavelength range: 630-770 nm) of micro-processed γ-$CsPbI_3$ thin film, using 458 nm excitation and a ×60/0.9NA objective. (B) Typical PL spectra corresponding on and off the micro-grid. (C) Lateral intensity which is averaged over the marked area in (A), showing no clear drop in intensity near the micro-grid boundary. (D) Fluorescence lifetime image recorded using pulsed 470 nm laser light (20 MHz; 250 nW/cm^2) focused by a ×10/0.4NA objective, with detection made over 650-750 nm wavelength range. (E) Distribution of lifetimes recorded over the mapped area, and (F) example of lifetime decay trace acquired from a large mapped area, with bi-exponential decay components. The inset displays the average results for bi-exponential decay fitting for data acquired from the center (inner 20 μm square) and border (8 μm from barrier) of 10 different squares. No detectable quenching of the PL intensity or lifetime near the micro-grid border is found. All measurements were performed under ambient conditions (~45%RH and 23°C).

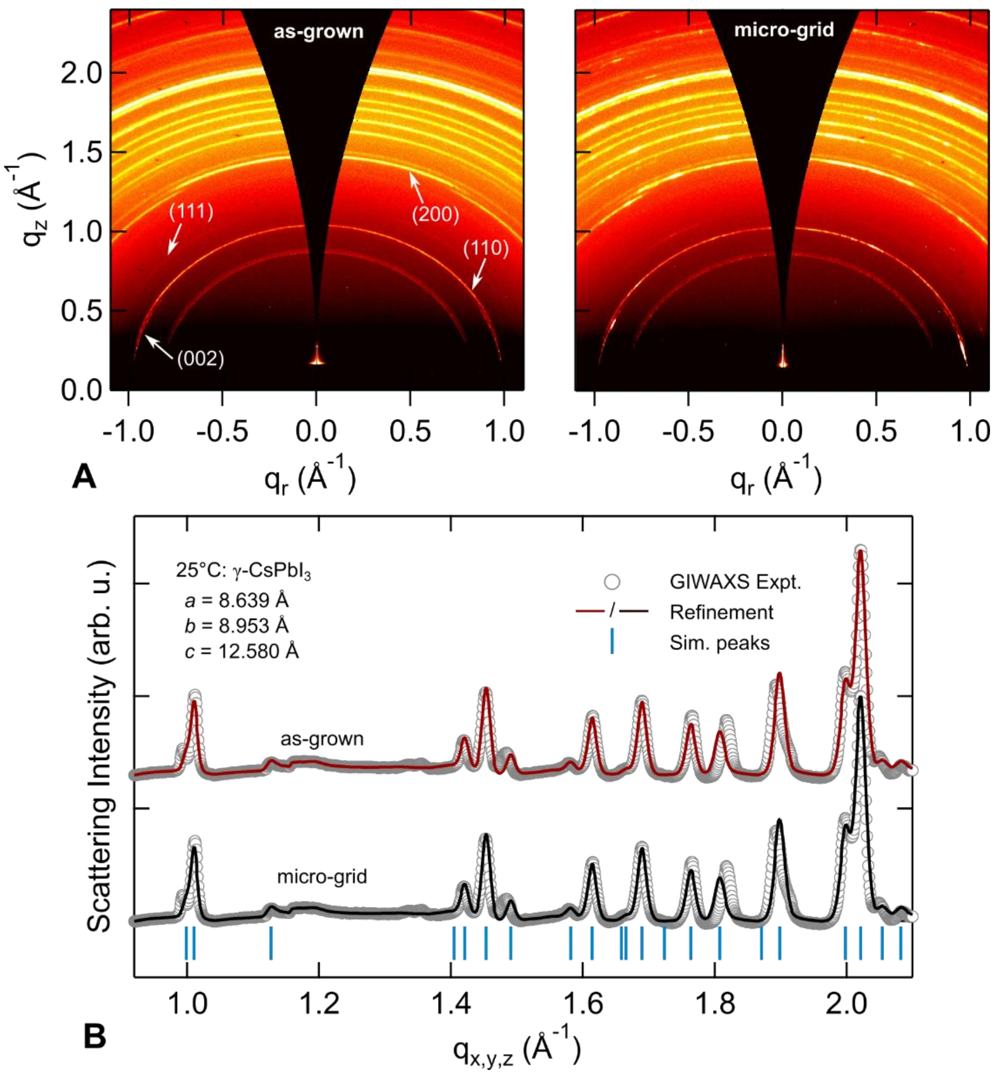

Fig. S38.

(A) 2D GIWAXS patterns and (B) corresponding integrated scattering signal (q_{xyz}) of as-grown and processed black $CsPbI_3$ thin films (under N_2). Structural refinement (Le Bail method) confirms their common orthorhombic perovskite phase, while the 2D images show the preferred orientation differences within the polycrystalline film, due to a change in anisotropy.

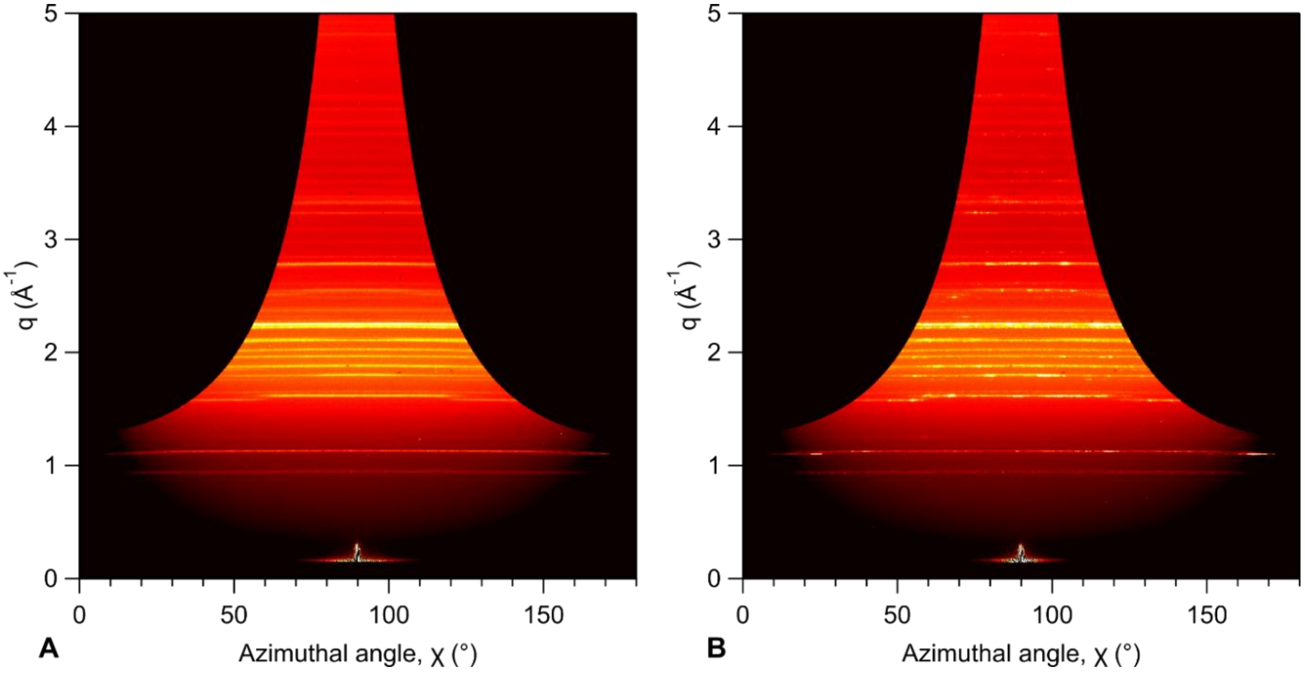

Fig. S39.

2D intensity images of (A) the thermally quenched strained black $CsPbI_3$ thin film and (B) the micro-processed sample, covering the complete set of diffraction rings as a linear function of the azimuthal angle.

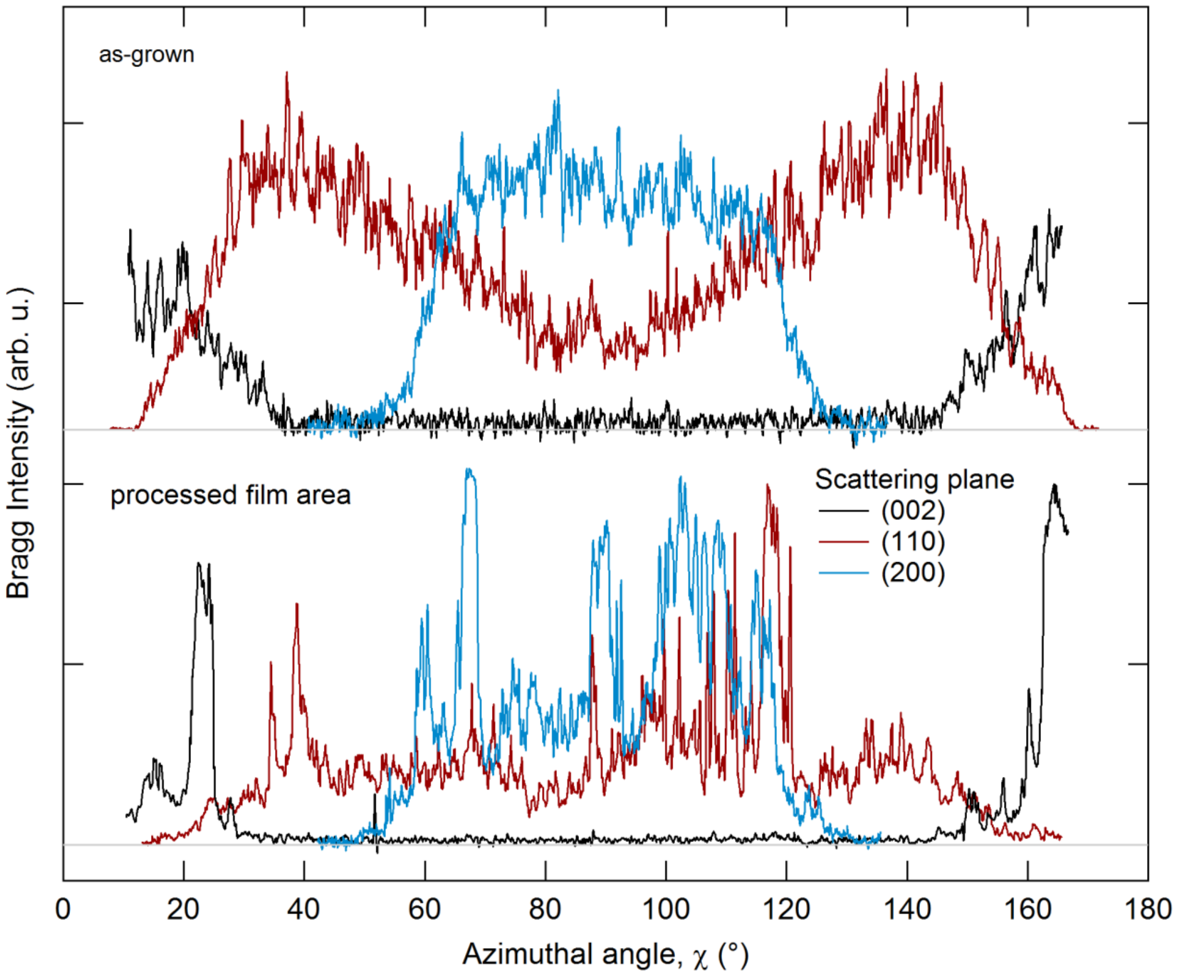

Fig. S40.

Normalized integrated intensity profiles of the (200), (110) and (002) peaks shown in Fig. S36 as a function of the azimuthal angle.

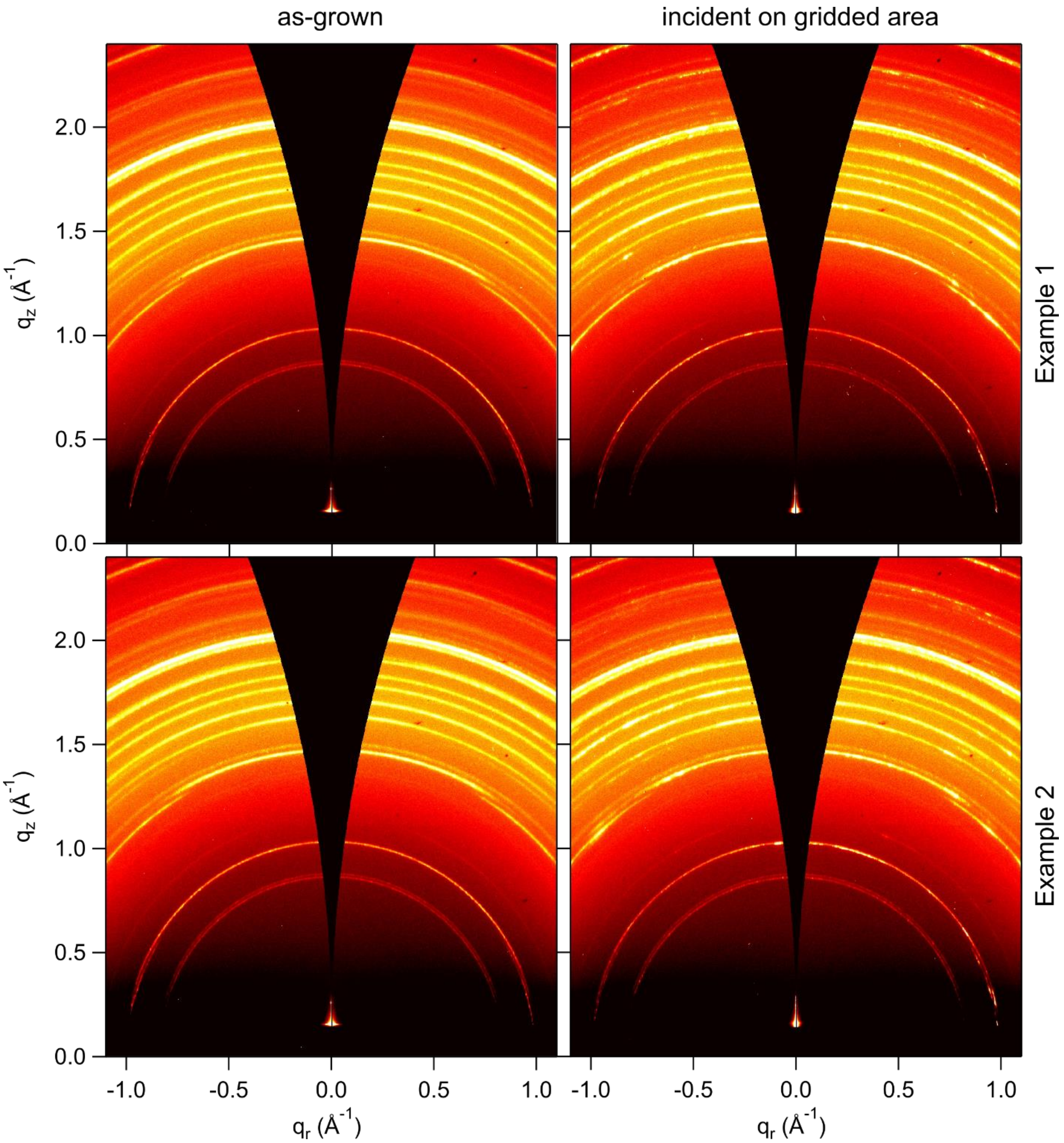

Fig. S41.

Synchrotron-based 2D GIWAXS patterns measured on two additional processed samples, both on and off the micro-patterned areas (40 µm) shortly after thermal quenching (under N_2).

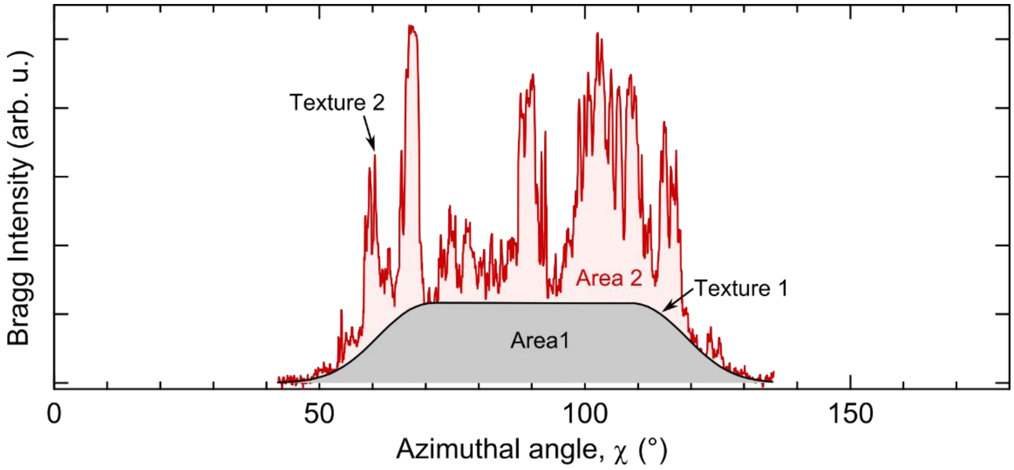

Fig. S42.

GIWAXS intensity of (200) γ-$CsPbI_3$ scattering planes measured with a 40 µm microgrid, as a function of azimuthal angle (χ) in the $\boldsymbol{q}_z$ vs $\boldsymbol{q}_{x,y}$ image of reciprocal space. Here we evaluate the relative x-ray scattering contributions arising from the overlap of Textures 1 and 2 recorded from a polycrystalline, black $CsPbI_3$ thin film. The GIWAXS beam H×V profile is roughly 50×100 µm at an incident angle of 1°, meaning that the incident footprint (several millimeters) encompasses many hundreds of grids. For a given azimuthal angle range, the area under the curve is assumed to be proportional to the scattering volume of crystal domains contributing to that particular texture expression. Texture 1 is shown to be an intrinsic expression of thermally treated $CsPbI_3$ thin film atop glass. Thus, subtracting the Area 1 lineshape from Area 2 we can estimate the relative population of grains influenced to additionally express Texture 2 (introduced by the microgrid). Through this analysis we arrive at the relative ratio for Texture 1 and 2 of approximately 3:1, which is consistent across multiple samples processed with 40 µm grids. Note that Texture 2 will average out to Texture 1 with a large enough beam size. This value thus represents the lower bound, with the true value being possibly higher.

Supplementary Note 8. Ambient processed and stable $CsPbI_3$ photodetectors integrated with a microgrid

Photodetector architecture: Thin film photodetectors we developed using a typical device architecture: ITO/NiO_x/$CsPbI_3$ (220 nm)/PCBM/BCP/Au. Here, an inverted *p-i-n* type device architecture was chosen with NiOx acting as the hole transport layer (HTL) and the phenyl-C 61-butyric acid methyl ester /bathocuproine (PCBM/BCP) acting as the electron transport layers (ETL), respectively. Typically the HTL in inorganic perovskite devices must be able withstand high thermal treatments required to convert the $CsPbI_3$ into the black-phase (>320 $^\circ$C), which mandates the use of inorganic metal oxide based HTLs. Among the several thermally stable metal oxide HTLs with good electrical properties reported thus far, NiO_x based perovskite devices have led directly to progress in the field. Moreover, the combination of NiO_x HTL and PCBM/BCP ETL are now well understood for their excellent charge extraction properties, and are widely used across several different types of perovskite devices (*4–6*). Here the requirement for a thermally robust HTL (due to high $CsPbI_3$ processing temperature) also means it is compatible with laser patterning, whereby the integrated microgrid imposes no limitations on the device, i.e. beyond the thermal processing limits already imposed by using an active $CsPbI_3$ perovskite layer. For the mixed halide device (Fig. S42), the same configuration was used with a perovskite layer which was grown with partial substitution of $PbBr_2$ in the precursor solution.

Microprocessing of the thin films via photolithography was found to be easily controlled and highly reproducible. We noticed that the most impactful variable for the performance of devices was in fact the inherent quality of the perovskite film, formed largely during steps such as spin coating of the $CsPbI_3$ film and deposition of the device carrier transport layers. This is because generating high-quality films consistently is a general issue for solution-processed perovskite materials, i.e. being influenced by factors like the chemist involved and environmental quality of the glovebox, or even the time of year (i.e. humidity and temperature). The $CsPbI_3$ perovskite phase of solution processed films are extremely unstable and some batches of films, for example, possessed excess pin holes and defects. The presence/absence of such features is able to trigger premature yellow phase nucleation and was a main source of variably, and placed limitations on our ability to produce consistent devices (Table S1).

Photolithographic patterning of photodetector: The same processing parameters are used in all devices to pattern a 2×2 cm^2 microgrid area of a wafer; 40 μm grid size, 458 nm laser excitation with 300 kW/cm^2 power and a scanning speed of 10 mm/s. A photograph of such a processed thin film device is shown in Fig. 4B of the main text, along with a SEM image.

Device characterization:: Devices tested in an ambient environment we simply stored, exposed to the normal laboratory conditions (i.e. 45-50% RH). Devices tested in a nitrogen atmosphere (Fig. S44) we stored in a glovebox and transferred for characterization once a day through an isolated connection.

Table S1.

Performance metrics of stabilized $CsPbI_3$ photodetector devices fashioned from different batches of solution processed films. All films have been stabilized with a microgrid and tested under 1 sun at an operating voltage of -0.5 V.

		Performance at 680 nm			Transient photocurent	
	Batch	EQE	R	D*	Rise (us)	Fall (us)
1	A1	29.66	0.163	2.50E+10	2.04	1.96
2	A2	27.47	0.151	4.46E+09	1.85	2.08
3	A3	30.50	0.167	3.58E+10	2.02	2.02
4	B1	29.21	0.160	1.24E+10	2.19	2.25
5	B2	27.57	0.151	4.08E+10	2.75	2.33
6	B3	27.40	0.150	3.81E+10	2.28	2.40
7	C1	28.96	0.159	2.17E+09	1.91	3.98
8	C2	31.16	0.171	6.45E+09	1.88	4.06
9	D1	30.16	0.165	7.51E+11	-	-
10	D2	29.61	0.162	1.49E+10	1.88	2.16
11	D3	30.59	0.168	1.03E+11	-	-
12	D4	30.69	0.168	1.82E+10	1.80	1.92

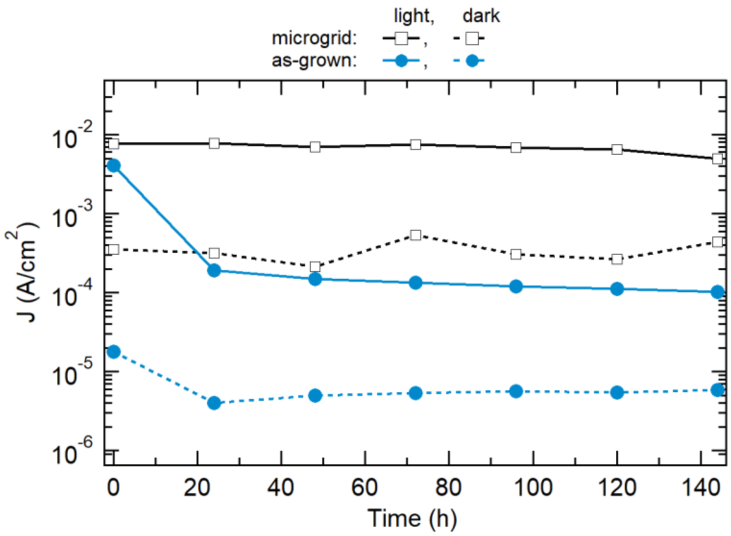

Fig. S43.

Tracking of dark and light (1 sun; -0.5 V) current for pure $CsPbI_3$ devices kept in an ambient atmosphere (45–50% RH) for one week.

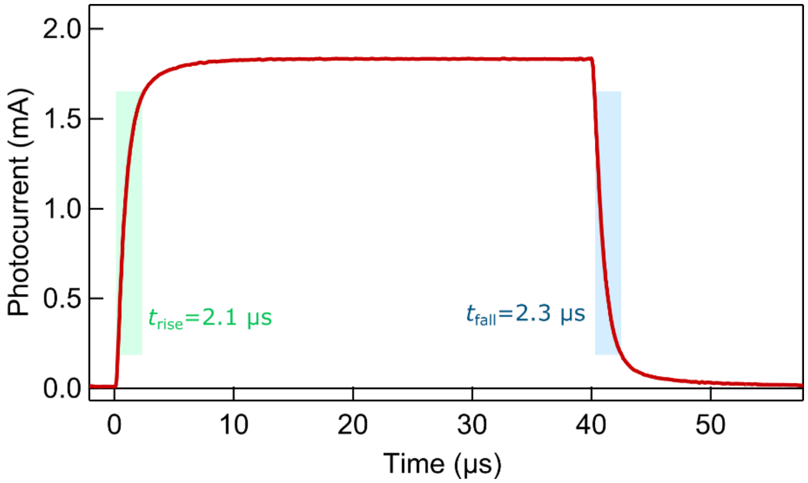

Fig. S44.

Rising and falling edges of one response cycle (~1 sun, 100 mW/cm^2) of a stabilized microprocessed $CsPbI_3$ photodetectors operated at -0.5 V. The response time of the photocurrent is inset, which is equivalent to the response speed of the device. It is defined as the time required for the photodetector output signal to change from 10% to 90% of the peak level.

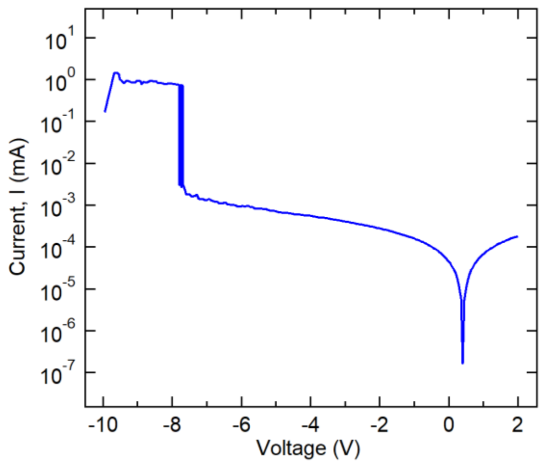

Fig. S45.

I-V characteristics of $CsPbI_3$ microgrid photodetector under a relatively large reverse bias, i.e. cathode positive with respect to the anode. The device continues to block current flow through it until the breakdown voltage point near -8 V, resulting in a sudden increase in reverse current.

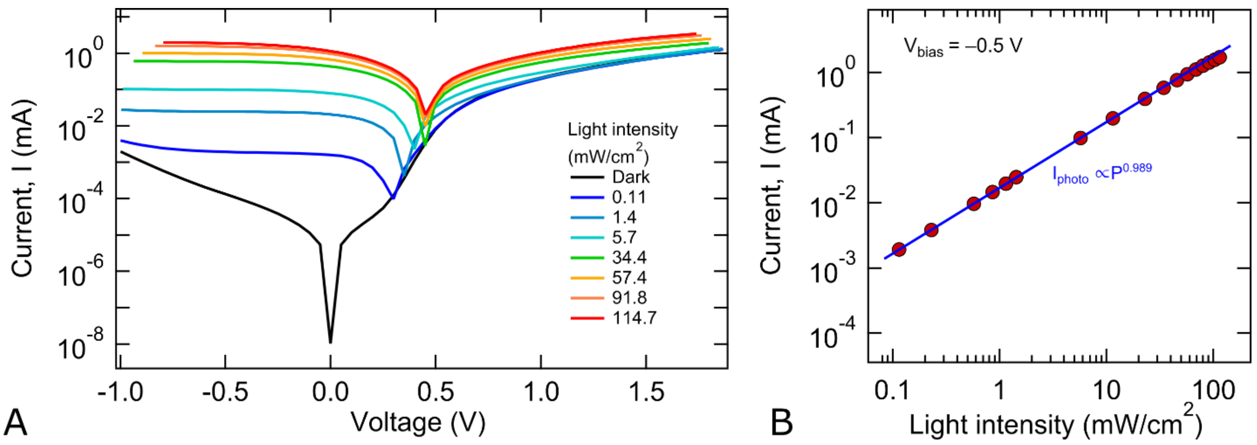

Fig. S46.

Optoelectronic characteristics of the $CsPbI_3$ microgrid photodetector at -0.5 V. (A) I–V curves of the photodetector under different light intensities. (B) Photocurrent under different incident light intensities. The fitting relationship between the photocurrent and the light intensity is inset, indicating the presence of low trap states, i.e. exponent ~1. At higher light intensity roll off is observed.

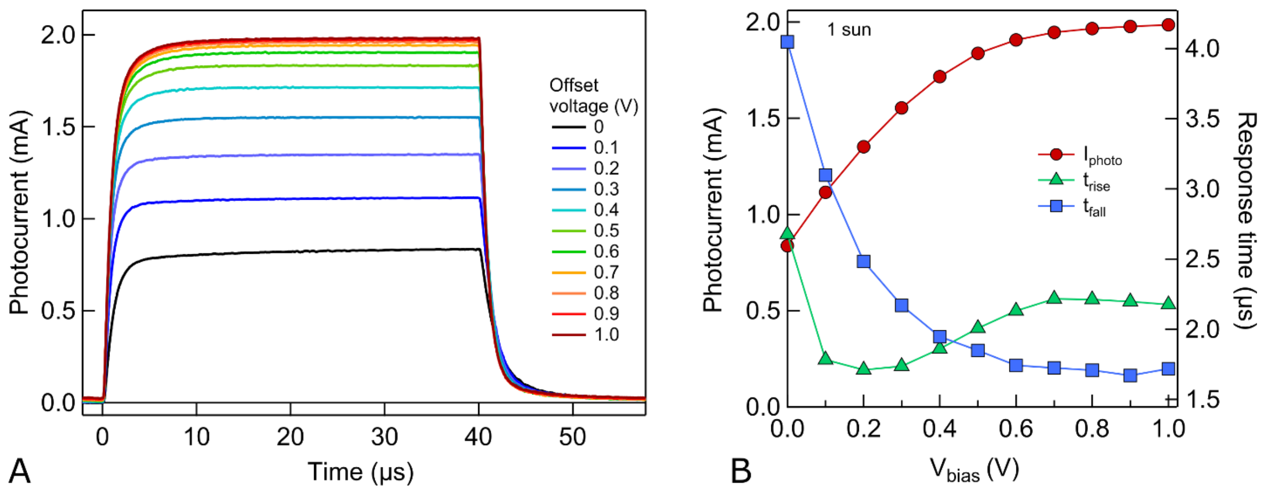

Fig. S47.

(A) Rising and falling edges of one response cycle (~1 sun, 100 mW/cm^2) of a microprocessed $CsPbI_3$ photodetectors under different offset voltages. (B) The photocurrent (I_{photo}) and response times (rise and fall) of the pulsed signal as a function of the bias voltage. Here we see saturation of the photocurrent beyond roughly 0.5 V and a stable decrease it the response times.

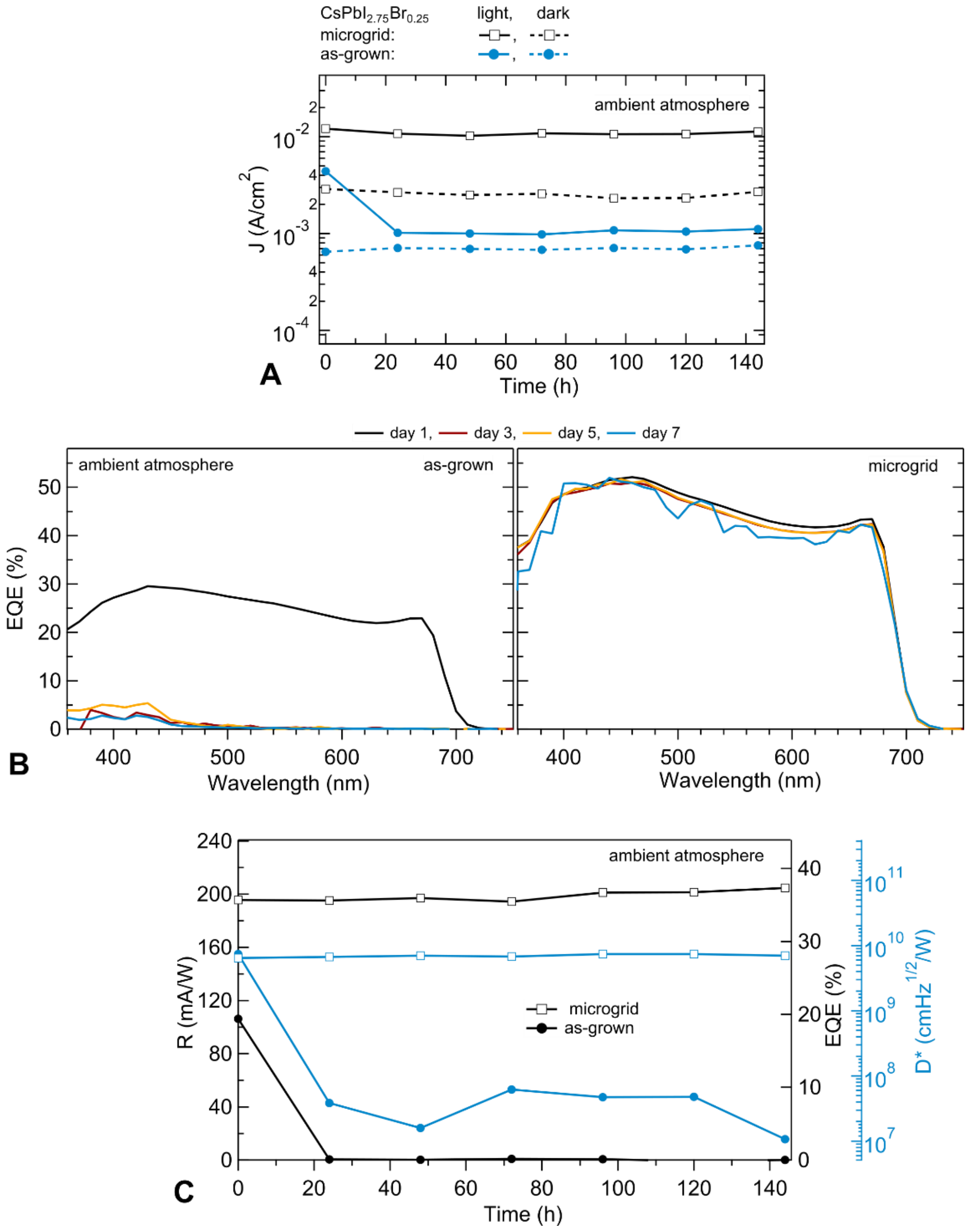

Fig. S48.

Tracking of device characteristics of mixed halide $CsPbI_{2.75}Br_{0.25}$ perovskite devices fitted with and without a 40 µm microgrid, and kept in an unregulated ambient atmosphere (45–50% RH) for one week. (A) dark and light (1 sun; -1.5 V) current, (B) EQE and (D) figures of merit (operating at 690 nm and -1.5 V) EQE, responsivity (*R*) and specific detectivity (*D**).

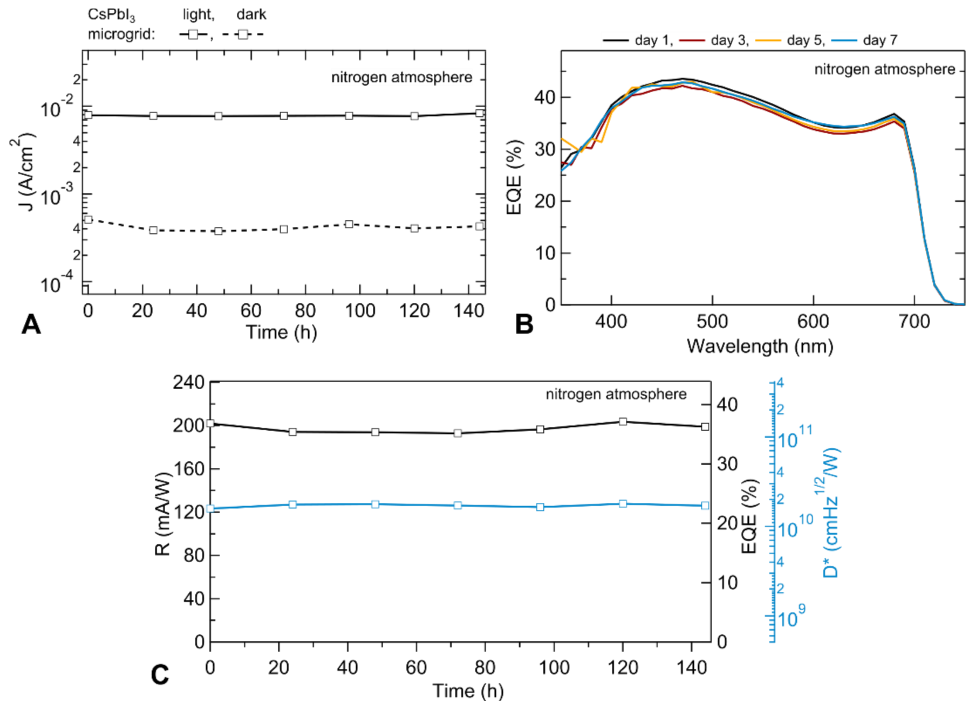

Fig. S49.

Tracking of device characteristics of pure $CsPbI_3$ perovskite devices fitted with a 40 μm microgrid and kept in a nitrogen atmosphere for one week. (A) dark and light (1 sun; -0.5 V) current, (B) EQE and (D) figures of merit (operating at 690 nm and -0.5 V) EQE, responsivity (*R*) and specific detectivity (*D**).

Supplementary References

1. J. A. Steele, H. Jin, I. Dovgaliuk, R. F. Berger, T. Braeckevelt, H. Yuan, C. Martin, E. Solano, K. Lejaeghere, S. M. J. Rogge, C. Notebaert, W. Vandezande, K. P. F. Janssen, B. Goderis, E. Debroye, Y.-K. Wang, Y. Dong, D. Ma, M. Saidaminov, H. Tan, Z. Lu, V. Dyadkin, D. Chernyshov, V. Van Speybroeck, E. H. Sargent, J. Hofkens, M. B. J. Roeffaers, Thermal unequilibrium of strained black $CsPbI_3$ thin films. *Science*. **365**, 679–684 (2019).

2. B. Zhao, S.-F. Jin, S. Huang, N. Liu, J.-Y. Ma, D.-J. Xue, Q. Han, J. Ding, Q.-Q. Ge, Y. Feng, J.-S. Hu, Thermodynamically Stable Orthorhombic γ-$CsPbI_3$ Thin Films for High-Performance Photovoltaics. *Journal of the American Chemical Society*. **140**, 11716–11725 (2018).

3. T. D. Kühne, M. Iannuzzi, M. Del Ben, V. V. Rybkin, P. Seewald, F. Stein, T. Laino, R. Z. Khaliullin, O. Schütt, F. Schiffmann, D. Golze, J. Wilhelm, S. Chulkov, M. H. Bani-Hashemian, V. Weber, U. Borštnik, M. Taillefumier, A. S. Jakobovits, A. Lazzaro, H. Pabst, T. Müller, R. Schade, M. Guidon, S. Andermatt, N. Holmberg, G. K. Schenter, A. Hehn, A. Bussy, F. Belleflamme, G. Tabacchi, A. Glöß, M. Lass, I. Bethune, C. J. Mundy, C. Plessl, M. Watkins, J. VandeVondele, M. Krack, J. Hutter, CP2K: An electronic structure and molecular dynamics software package - Quickstep: Efficient and accurate electronic structure calculations. *J. Chem. Phys.* **152**, 194103 (2020).

4. K. C. Wang, P. S. Shen, M. H. Li, S. Chen, M. W. Lin, P. Chen, T. F. Guo, Low-temperature sputtered nickel oxide compact thin film as effective electron blocking layer for mesoscopic $NiO/CH_3NH_3PbI_3$ perovskite heterojunction solar cells. *ACS Applied Materials and Interfaces*. **6**, 11851–11858 (2014).

5. J. Y. Jeng, K. C. Chen, T. Y. Chiang, P. Y. Lin, T. Da Tsai, Y. C. Chang, T. F. Guo, P. Chen, T. C. Wen, Y. J. Hsu, Nickel oxide electrode interlayer in $CH_3NH_3PbI_3$ perovskite/PCBM planar-heterojunction hybrid solar cells. *Advanced Materials*. **26**, 4107–4113 (2014).

6. A. M. Afzal, I. G. Bae, Y. Aggarwal, J. Park, H. R. Jeong, E. H. Choi, B. Park, Highly efficient self-powered perovskite photodiode with an electron-blocking hole-transport NiO_x layer. *Scientific Reports*. **11**, 169 (2021).
